# A Stable and Scalable Digital Composite Neurocognitive Test for Early Dementia Screening Based on Machine Learning: Model Development and Validation Study

**DOI:** 10.2196/49147

**Published:** 2023-12-01

**Authors:** Dongmei Gu, Xiaozhen Lv, Chuan Shi, Tianhong Zhang, Sha Liu, Zili Fan, Lihui Tu, Ming Zhang, Nan Zhang, Liming Chen, Zhijiang Wang, Jing Wang, Ying Zhang, Huizi Li, Luchun Wang, Jiahui Zhu, Yaonan Zheng, Huali Wang, Xin Yu, Alzheimer's Disease Neuroimaging Initiative (ADNI)

**Affiliations:** 1 Clinical Research Division Dementia Care and Research Center Peking University Institute of Mental Health (Sixth Hospital) Beijing China; 2 Beijing Dementia Key Lab National Clinical Research Center for Mental Disorders (Peking University) National Health Committee Key Laboratory of Mental Health Beijing China; 3 Shanghai Key Laboratory of Psychotic Disorders Shanghai Mental Health Center Shanghai Jiao Tong University School of Medicine Shanghai China; 4 Department of Psychiatry First Hospital of Shanxi Medical University Taiyuan China; 5 Beijing Key Laboratory of Mental Disorders National Clinical Research Center for Mental Disorders Beijing Anding Hospital, Capital Medical University Beijing China; 6 Department of Psychiatry The Third Afﬁliated Hospital of Sun Yat-sen University Guangzhou China; 7 Department of Neurology Tianjin Medical University General Hospital Tianjin China; 8 China Telecom Digital Intelligence Technology Co.,Ltd Beijing China; 9 see Acknowledgements

**Keywords:** mild cognitive impairment, digital cognitive assessment, machine learning, neurocognitive test, cognitive screening, dementia

## Abstract

**Background:**

Dementia has become a major public health concern due to its heavy disease burden. Mild cognitive impairment (MCI) is a transitional stage between healthy aging and dementia. Early identification of MCI is an essential step in dementia prevention.

**Objective:**

Based on machine learning (ML) methods, this study aimed to develop and validate a stable and scalable panel of cognitive tests for the early detection of MCI and dementia based on the Chinese Neuropsychological Consensus Battery (CNCB) in the Chinese Neuropsychological Normative Project (CN-NORM) cohort.

**Methods:**

CN-NORM was a nationwide, multicenter study conducted in China with 871 participants, including an MCI group (n=327, 37.5%), a dementia group (n=186, 21.4%), and a cognitively normal (CN) group (n=358, 41.1%). We used the following 4 algorithms to select candidate variables: the *F*-score according to the SelectKBest method, the area under the curve (AUC) from logistic regression (LR), *P* values from the logit method, and backward stepwise elimination. Different models were constructed after considering the administration duration and complexity of combinations of various tests. Receiver operating characteristic curve and AUC metrics were used to evaluate the discriminative ability of the models via stratified sampling cross-validation and LR and support vector classification (SVC) algorithms. This model was further validated in the Alzheimer’s Disease Neuroimaging Initiative phase 3 (ADNI-3) cohort (N=743), which included 416 (56%) CN subjects, 237 (31.9%) patients with MCI, and 90 (12.1%) patients with dementia.

**Results:**

Except for social cognition, all other domains in the CNCB differed between the MCI and CN groups (*P*<.008). In feature selection results regarding discrimination between the MCI and CN groups, the Hopkins Verbal Learning Test-5 minutes Recall had the best performance, with the highest mean AUC of up to 0.80 (SD 0.02) and an *F*-score of up to 258.70. The scalability of model 5 (Hopkins Verbal Learning Test-5 minutes Recall and Trail Making Test-B) was the lowest. Model 5 achieved a higher level of discrimination than the Hong Kong Brief Cognitive test score in distinguishing between the MCI and CN groups (*P*<.05). Model 5 also provided the highest sensitivity of up to 0.82 (range 0.72-0.92) and 0.83 (range 0.75-0.91) according to LR and SVC, respectively. This model yielded a similar robust discriminative performance in the ADNI-3 cohort regarding differentiation between the MCI and CN groups, with a mean AUC of up to 0.81 (SD 0) according to both LR and SVC algorithms.

**Conclusions:**

We developed a stable and scalable composite neurocognitive test based on ML that could differentiate not only between patients with MCI and controls but also between patients with different stages of cognitive impairment. This composite neurocognitive test is a feasible and practical digital biomarker that can potentially be used in large-scale cognitive screening and intervention studies.

## Introduction

### Background

Dementia is currently a major public health problem and one of the major causes of disability in older people [1,2]. Mild cognitive impairment (MCI) involves abnormal cognitive function in 1 or more cognitive domains without the loss of functional abilities and skills for everyday life [3]. It represents a transitional stage between healthy aging and dementia and affects 10%-15% of the population over the age of 65 years [4]. Early detection of MCI and identification of modifiable risk factors could profoundly reduce the prevalence of MCI and subsequent dementia [5].

Current clinical biomarkers of cerebral amyloid and tau protein deposition that rely on positron emission tomography (PET) and cerebrospinal fluid (CSF) are invasive and expensive. The use of these biomarkers to detect dementia in large populations remains difficult [6]. Paper-and-pencil-based cognitive tests remain the most commonly used first-line screening tools for MCI and dementia [6,7]. However, these tests need to be administered by trained assessors and are time-consuming [8]. The short duration of most primary care visits and the lack of formally trained assessors are the key barriers to large-scale assessment in the primary care setting [9]. Another challenge of most widely used cognitive tests for MCI screening is that their efficacy is compromised among populations with low levels of education or illiteracy [10]. Therefore, there is an urgent need for time saving, easily administered, and reliable cognitive tools to carry out large-scale cognitive screening.

### Objective

We previously developed the Chinese Neuropsychological Consensus Battery (CNCB) via the Delphi method; all tests in the CNCB are culturally appropriate and have been validated in Chinese individuals [11]. The CNCB covers 6 subdomains, including attention, memory, executive function, language, visuospatial function, and social cognition [11]. We further digitized this comprehensive cognitive battery such that it could be administered on a touchscreen computer [12]. The computerized CNCB is a comprehensive tool for the assessment of cognitive decline, but it is time-consuming to complete as it contains many tests. This study aimed to use machine learning (ML) in the Chinese Neuropsychological Normative Project (CN-NORM) cohort to develop a stable and scalable composite neurocognitive test based on the CNCB for the early detection of MCI and dementia. We also performed external validation with the Alzheimer’s Disease Neuroimaging Initiative phase 3 (ADNI-3) cohort, which is ethnically different from the CN-NORM cohort.

## Methods

### Study Design and Participants

CN-NORM was led by the Dementia Care & Research Center, Peking University Institute of Mental Health (Sixth Hospital), China. Participants were consecutively recruited for CN-NORM from August 28, 2019, to November 1, 2022. CN-NORM was a multicenter study conducted by 7 hospitals in China. As shown in Figure S1 in Multimedia Appendix 1, the final study population consisted of 871 participants, including the cognitively normal (CN) group (n=358, 41.1%), the MCI group (n=327, 37.5%), and the dementia group (n=186, 21.4%), between the ages of 55 and 85 years.

All participants had more than 5 years of education. The inclusion criteria for the MCI group were as follows: (1) met the *Diagnostic and Statistical Manual of Mental Disorders, Fifth Edition* (DSM-5) criteria for mild neurocognitive disorder (NCD) [13], (2) had preserved global cognitive function, (3) had intact or only mildly impaired daily living ability, and (4) did not meet any criteria for dementia. The inclusion criteria for the dementia group were as follows: (1) met the DSM-5 criteria for NCD [13] and (2) had a Clinical Dementia Rating score of 0.5-2. The inclusion criteria for the CN group were as follows: (1) did not meet the clinical criteria for cognitive impairment and (2) did not have memory or cognitive complaints or objective cognitive impairment. The exclusion criteria for all groups were as follows: (1) the presence of neurological or mental disorders that may affect cognitive function, such as schizophrenia or substance use disorders, or (2) the presence of major medical problems, such as cancer or cerebrovascular events.

### ADNI-3 Cohort

The ADNI-3 cohort was used for validation. The data used in the preparation of this paper were partly obtained from the ADNI database [14]. The ADNI was launched in 2003 as a public-private partnership, led by Principal Investigator Michael W Weiner, MD. The primary goal of the ADNI was to test whether serial magnetic resonance imaging (MRI), PET, other biological markers, and clinical and neuropsychological assessments can be combined to measure the progression of MCI and early Alzheimer’s disease (AD). The ADNI was approved by the Institutional Review Boards of all participating institutions, and written informed consent was obtained from all participants at each site.

According to our study aim, we selected subjects in the ADNI phase 3 (ADNI-3) who completed both the Trail Making Test-B and the 10-word delayed recall test from the Alzheimer’s Disease Assessment Scale-Cognitive (ADAS-Cog). Participants were included consecutively. After data preprocessing and removal of invalid records, the cohort included 743 individuals: 416 (56%) CN individuals, 237 (31.9%) patients with MCI, and 90 (12.1%) patients with dementia. The CN group showed no signs of depression, MCI, or dementia. Experienced neurologists or psychiatrists determined the best diagnosis (CN, MCI, dementia) based on the results of clinical, neuropsychological, and laboratory information. The diagnosis was also reviewed and confirmed by the Central Review Committee in the ADNI. The MCI group included patients with amnestic and nonamnestic MCI.

### Neuropsychological Assessment

Global cognitive function was evaluated with the Hong Kong Brief Cognitive (HKBC) test, which is a pen-and-paper cognitive test. The HKBC test was developed for older people with a lower education level and covers multiple cognitive domains [15]. Moreover, the HKBC test has been further validated for identifying patients with amnestic MCI or dementia in a Chinese population [16]. Among cognitive screening tools, the HKBC test has the highest validity and reliability in identifying the earliest stages of subtle cognitive decline [8,15]. Thus, the HKBC test was used as the reference cognitive assessment tool in this study.

The neurocognitive function of all participants in the CN-NORM cohort was assessed using the CNCB [11]. Both the Trail Making Test-B and the 10-word delayed recall test from the ADAS-Cog were selected for model validation in the ADNI-3. The specific content of the CNCB and cognitive tests in the ADNI-3 cohort are shown in Table S1 in Multimedia Appendix 1 and the *Method* section in Multimedia Appendix 1. Details of the assessment of depressive symptoms are shown in the *Method* section in Multimedia Appendix 1.

All raw scores of the cognitive battery were adjusted for the demographic predictors of sex, age, and education. Specifically, in the CN group, cognitive test results were used as outcomes in linear regression models with sex, age, and years of education as predictors (included if significant). The result was converted to a *z*-score based on the test score distribution in the present population. The following equation was used to calculate the *z*-score:

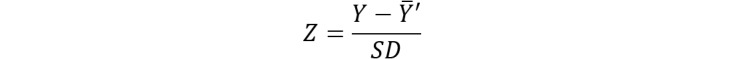



where Z is the *z*-score estimate for an individual subject, Y is the raw score for an individual subject obtained from the performance on a given test, 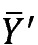 is the predicted population mean score, and SD is the standard deviation, which we substitute as the CN group’s SD.

The model intercept, estimates, and root SD from the models in the CN group were then applied to the cognitive test results in both CN-NORM and ADNI-3 cohorts to calculate cognitive test *z*-scores, as reported by Palmqvist et al [17], Borland et al [18], and Shirk et al [19].

### Data Selection and Preprocessing

The flowchart of participant recruitment for the CN-NORM cohort is shown in Figure S1 in Multimedia Appendix 1. After application of inclusion criteria and integrity filtering, 871 participants were retained and included in the analysis. Most missing data were from the MCI or CN group, who were capable of completing the tests. However, due to computer recording, equipment problems, or the subjects being tired and not completing the tests, these data were missing. The mean value method was used to fill in the missing data.

### Statistical Analysis

The characteristics of all participants were summarized using descriptive statistics. Sex and marital status were analyzed with the chi-square (*χ*^2^) test. Comparisons of continuous data results among the 3 groups were analyzed using the nonparametric (Kruskal-Wallis) test as distributions of those variables were not normal. The distributions of these continuous data are listed in Table S2 in Multimedia Appendix 1. *P* values were compared against a Bonferroni-adjusted value.

#### Feature Selection and Model Development

**Figure 1:**
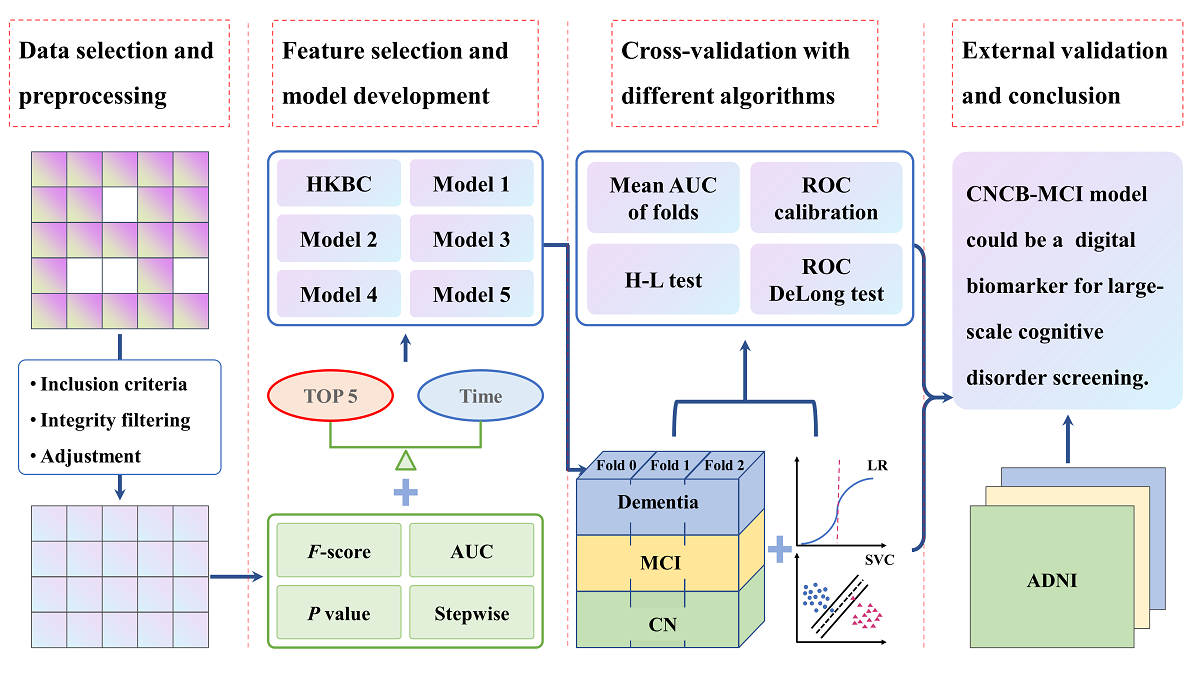
The algorithm pathway of the brief panel for MCI screening. ADNI: Alzheimer’s Disease Neuroimaging Initiative; AUC: area under the curve; CN: cognitively normal; CNCB: Chinese Neuropsychological Consensus Battery; HKBC: Hong Kong Brief Cognitive; H-L, Hosmer-Lemeshow; LR: logistic regression; MCI: mild cognitive impairment; ROC: receiver operating characteristic; SVC: support vector classification.

The algorithm pathway for selecting optimal ML models is shown in Figure 1. There were 31 primary variables derived from 24 cognitive tests in the CNCB. Based on each test *z*-score, we used the following 4 algorithms to perform feature selection: the *F*-score according to the SelectKBest method, the area under the curve (AUC) according to the logistic regression (LR) algorithm, the *P* value according to the logit method, and backward stepwise elimination. Specifically, the top 5 variables in terms of both the *F*-score and the AUC that discriminated the MCI group from the CN group were selected for inclusion in candidate models. Different models were constructed after considering the administration duration and complexity of combinations of various models.

#### Evaluation and Analysis

The performance of each model was assessed in terms of discrimination and calibration. Receiver operating characteristic (ROC) analysis was used to evaluate the discrimination ability of different models via LR and support vector classification (SVC) with a linear kernel algorithm. The regularization parameter C in SVC was set to 0.5. To preserve the distribution of classes in each split, a stratified 3-fold cross-validation strategy was used to develop and validate the models [20]. The mean AUC value was calculated based on 3 folds. The validation set (1 fold of data) was set aside and used only for evaluation. The DeLong test was performed to compare the ROC curves among each model and HKBC test scores. Calibration curves were used to assess the calibration of predictions of a binary classifier. The Hosmer-Lemeshow (H-L) test was used to determine the *χ*^2^ goodness of fit of each model. A 2-sided *P* value of <.05 was considered statistically significant. Python (v3.8), scikit-learn (v0.24.1), scipy (v1.5.2), statsmodels (v0.13.2), matplotlib (v3.3.4), seaborn (v0.11.1), and SPSS version 20.0 (IBM) were used for data analysis and visualization.

### Ethical Considerations

CN-NORM was approved by the Ethics Committee at Peking University Sixth Hospital—approval number: (2019) Ethics (No.4). All participants provided written informed consent before participation. They were not compensated for their participation, and they were informed of this in the informed consent form. Concerning data protection and confidentiality, personal information was labeled in a nonpersonally identifiable way.

## Results

### Characteristics of Participants

The characteristics of the participants in the CN-NORM and ADNI-3 cohorts are shown in Tables 1 and 2. Significant differences were observed in age, sex, and the Geriatric Depressive Scale (GDS) score (but not education level) among the CN, MCI, and dementia groups (*P*<.05) in the CN-NORM cohort, while significant differences were found in all variables (sex, education level, marital status, GDS score, and age) among the 3 groups (*P*<.05) in the ADNI-3 cohort.

**Table 1:** Demographic characteristics of participants from the CN-NORM^a^ (N=871) cohort.

Characteristics	CN^b^ group (n=358)	MCI^c^ group (n=327)	Dementia group (n=186)	H^d^/*χ*^2^ (*df*)^e^	*P* value
Female, n (%)	186 (52.0)	215 (65.7)	113 (60.8)	13.74 (2)	.001
Age (years), mean (SD)	68.8 (5.4)	72.0 (7.2)	74.8 (7.2)	97.56	<.001
Education (years), mean (SD)	11.9 (3.2)	12.3 (3.4)	12.4 (3.4)	5.45	.07
Single, divorced, or widowed, n (%)	40 (12.1)	49 (15.0)	34 (18.3)	7.62 (2)	.06
GDS, mean (SD)	3.3 (2.7)^f^	6.2 (4.7)^f^	6.7 (5.2)^f^	54.73	<.001

^a^CN-NORM: Chinese Neuropsychological Normative Project.

^b^CN: cognitively normal.

^c^MCI: mild cognitive impairment.

^d^Effect size of the Kruskal-Wallis test.

^e^Age, education, and the Geriatric Depressive Scale (GDS) score were analyzed using the nonparametric (Kruskal-Wallis) test as the distributions of these variables were not normal. The chi-square (*χ*^2^) test was used to analyze sex and marital status.

^f^30 items.

**Table 2:** Demographic characteristics of participants from the ADNI-3^a^ (N=743) cohort.

Characteristics	CN^b^ group (n=416)	MCI^c^ group (n=237)	Dementia group (n=90)	H^d^/*χ*^2^ (*df*)^e^	*P* value
Female, n (%)	242 (58.2)	103 (43.5)	35 (38.9)	19.24 (2)	<.001
Age (years), mean (SD)	73.7 (6.7)	74.8 (6.4)	74.8 (7.0)	6.03	.049
Education (years), mean (SD)	16.8 (2.3)	16.0 (2.5)	15.8 (2.5)	20.67	<.001
Single, divorced, or widowed, n (%)	115 (27.6)	47 (19.8)	11 (12.2)	12.18 (2)	.002
GDS, mean (SD)	1.1 (1.4)^f^	2.4 (2.4)^f^	2.7 (2.3)^f^	98.21	<.001

^a^ADNI: Alzheimer’s Disease Neuroimaging Initiative phase 3.

^b^CN: cognitively normal.

^c^MCI: mild cognitive impairment.

^d^Effect size of the Kruskal-Wallis test.

^e^Age, education, and the Geriatric Depressive Scale (GDS) score were analyzed using the nonparametric (Kruskal-Wallis) test as the distributions of these variables were not normal. The chi-square (*χ*^2^) test was used to analyze sex and marital status.

^f^15 items.

### Feature Selection and Model Development

**Figure 2:**
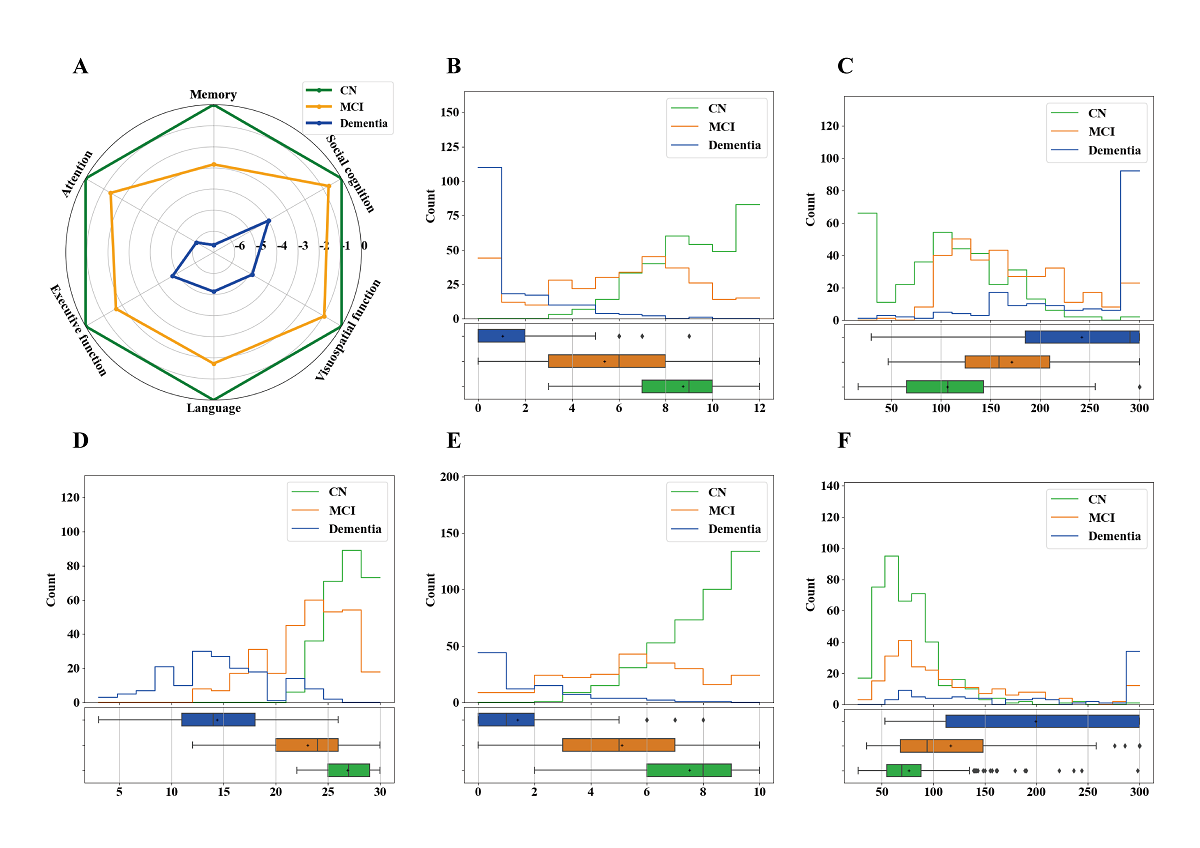
Cognitive profiles over six cognitive domains and part of variables data distribution among CN, MCI and dementia group. (A) Cognitive profiles over six cognitive domains by CNCB battery among CN, MCI and dementia group after adjusting for age, sex and education. (B) The variables data distribution of Hopkins Verbal Learning Test-5 minutes Recall by CNCB battery among CN, MCI and dementia group. (C) The variables data distribution of Trail Making Test-B by CNCB battery among CN, MCI and dementia group. (D) The variables data distribution of HKBC by CNCB battery among CN, MCI and dementia group. (E) The variables data distribution of ADAS-Cog word recall by ADNI-3 cohort among CN, MCI and dementia group. (F) The variables data distribution of Trail Making Test-B by ADNI-3 cohort among CN, MCI and dementia group. ADNI, Alzheimer’s Disease Neuroimaging Initiative; CBCB, Chinese Neuropsychological Consensus Battery; CN, Cognitive Normal Controls; HKBC, Hong Kong Brief Cognitive; MCI, Mild Cognitive Impairment.

The cognitive results of each group in the CN-NORM cohort are presented in Tables S3 and S4 in Multimedia Appendix 1. All variables’ raw and *z*-scores on the 24 cognitive tests in the CNCB differed among the CN, MCI, and dementia groups (*P*<.002). Except for the Digit Span-Backward Length Test, the Eye Emotional Recognition Task-Gender Test, and the Clock Drawing Test, scores of all variables differed between the CN and MCI groups (*P*<.002). Part of the comparison of variable data is shown in Figure 2. Except for social cognition, all other domains differed between the MCI and CN groups (*P*<.008), as shown in Figure 2A and Tables S3-S5 in Multimedia Appendix 1. Memory and executive function were the 2 cognitive domains most severely impaired in the MCI group, with the highest Cohen *d* (in descending order of impairment: memory > executive function > language > attention> visuospatial function > social cognition) as shown in Table S5 in Multimedia Appendix 1.

**Figure 3:**
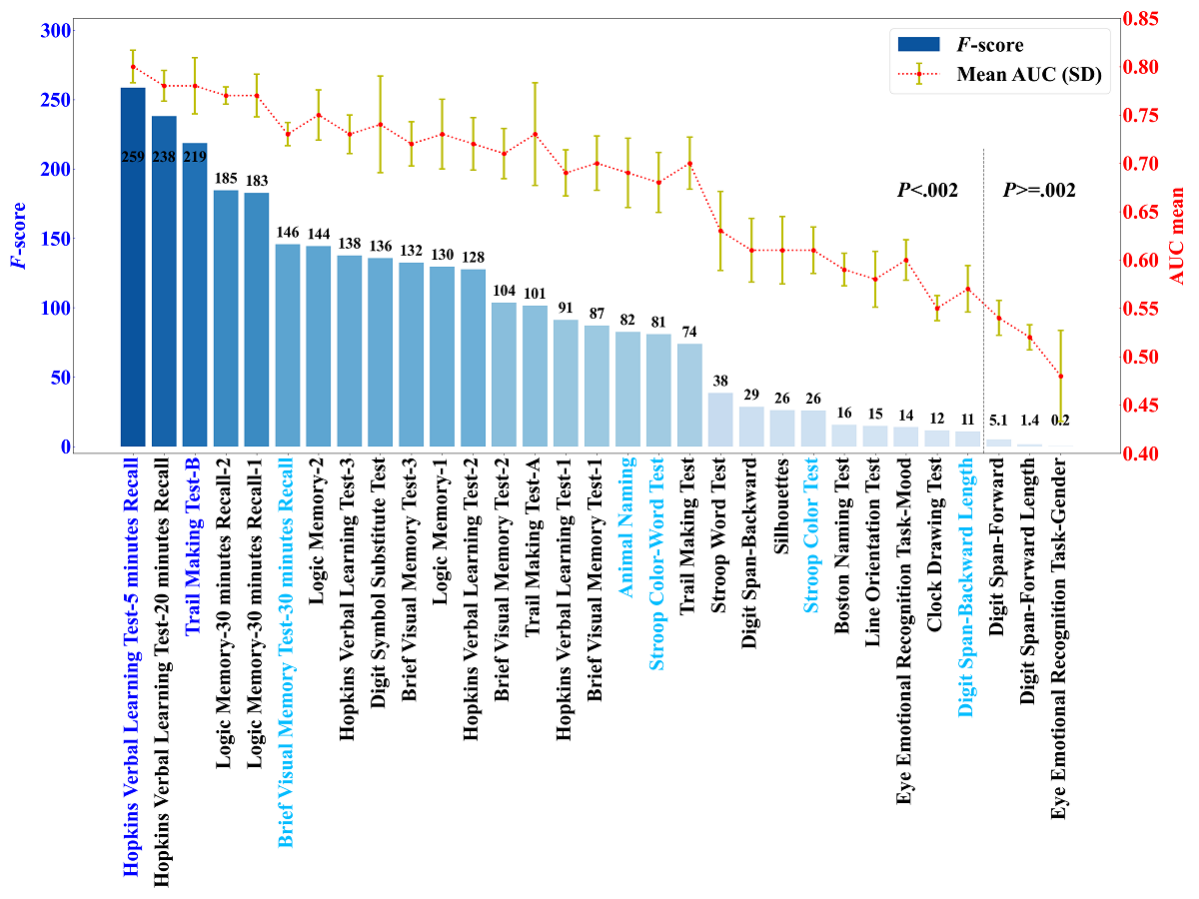
Feature selection results using different ML methods for distinguishing the MCI and CN groups in the CN-NORM cohort. The graph shows candidate variables’ selection results with SelectKBest using *F*-scores and *P* values, LR using the AUC, and backward stepwise elimination. Both dark- and light-blue colors are the results of backward stepwise elimination. The bar chart (left axis) represents the importance of variables based on SelectKBest with *F*-scores. The line chart (right axis) shows the importance of variables based on LR with the AUC. The top 5 variables were selected for ML model building. *P* values in feature selection were compared against a Bonferroni-adjusted value, given the number of tests per hypothesis: α=.05/number of tests (31), 0.002. AUC: area under the curve; CN: cognitively normal; CN-NORM: Chinese Neuropsychological Normative Project; MCI: mild cognitive impairment; LR: logistic regression; ML machine learning.

**Figure 4:**
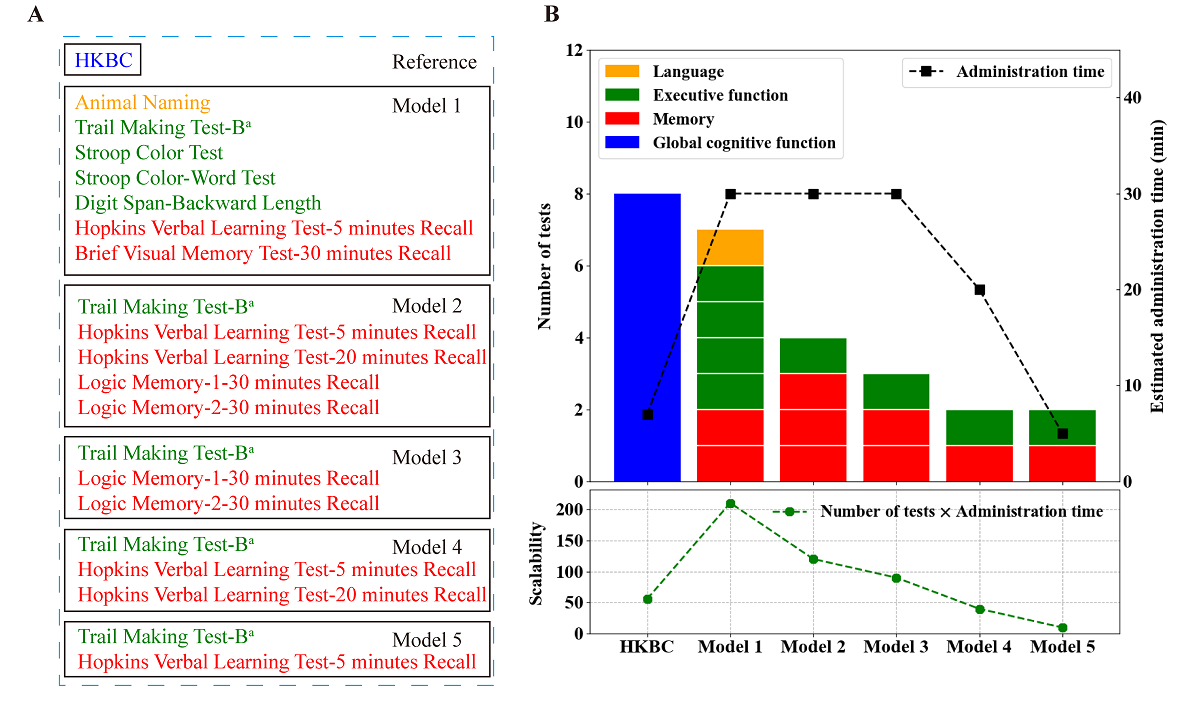
Description and comprehensive evaluation of scalability of different models for distinguish the MCI and CN groups. (A) Description of different models. (B) Comprehensive evaluation of scalability of different models. CN: cognitively normal; HKBC: Hong Kong Brief Cognitive; MCI: mild cognitive impairment.

The feature selection results regarding discrimination between the MCI and CN groups are shown in Figure 3 and Tables S6 and S7 in Multimedia Appendix 1. The Hopkins Verbal Learning Test-5 minutes Recall had the best performance, with the highest mean AUC of up to 0.80 (SD 0.02) and an *F*-score of up to 258.70. Details of 5 different models are listed in Figure 4. Model 5 was simplified from model 4, and it included only the Hopkins Verbal Learning Test-5 minutes Recall and the Trail Making Test-B. Variables in model 5 were also candidate variables with overlap on all 4 algorithms.

Based on the selection results, different models were constructed, as shown in Figure 4A. Seven variables (Animal Naming Test, Trail Making Test-B, Stroop Color Test, Stroop Color-Word Test, Digit Span-Backward Length Test, Hopkins Verbal Learning Test-5 minutes Recall, and Brief Visual Memory Test-30 minutes Recall) were included in model 1 through backward stepwise elimination (Figure 4 and Table S6 in Multimedia Appendix 1). Model 1 covered 3 domains: language, memory, and executive function (Figure 4B). The top 5 variables, selected using SelectKBest and the AUC LR methods, were similar (Table S7 in Multimedia Appendix 1 and Figure 3); the other variables declined sharply in discriminative power. Therefore, the top 5 results were selected for inclusion in model 2, which covered memory (Hopkins Verbal Learning Test-5 minutes Recall, Hopkins Verbal Learning Test-20 minutes Recall, Logic Memory-1-30 minutes Recall, Logic Memory-2-30 minutes Recall) and executive function (Trail Making Test-B), as shown in Figure 4A. Given the administration duration and complexity of combinations, these variables were further divided into different combinations. Model 3 was composed of semantic memory (Logic Memory Test) and executive function (Trail Making Test-B). Model 4 was composed of word memory (Hopkins Verbal Learning Test-5 minutes Recall and Hopkins Verbal Learning Test-20 minutes Recall) and execution function (Trail Making Test-B). Model 5 was simplified from model 4, considering time; it included only the Hopkins Verbal Learning Test-5 minutes Recall and Trail Making Test-B. Variables in model 5 were candidate variables with overlap on all 4 algorithms.

As the Trail Making Test-B could be completed in 5 minutes between the Hopkin’s Verbal Learning Test, it took approximately 5 minutes to complete model 5. To comprehensively evaluate the scalability of the different models, the number of tests was multiplied by the administration duration (number of tests × administration duration) to determine the scalability of the models, with a low value being better. The scalability of model 5 was the lowest, approximately 10 (2 × 5), as shown in Figure 4B.

### Internal Validation in the CN-NORM Cohort

**Figure 5:**
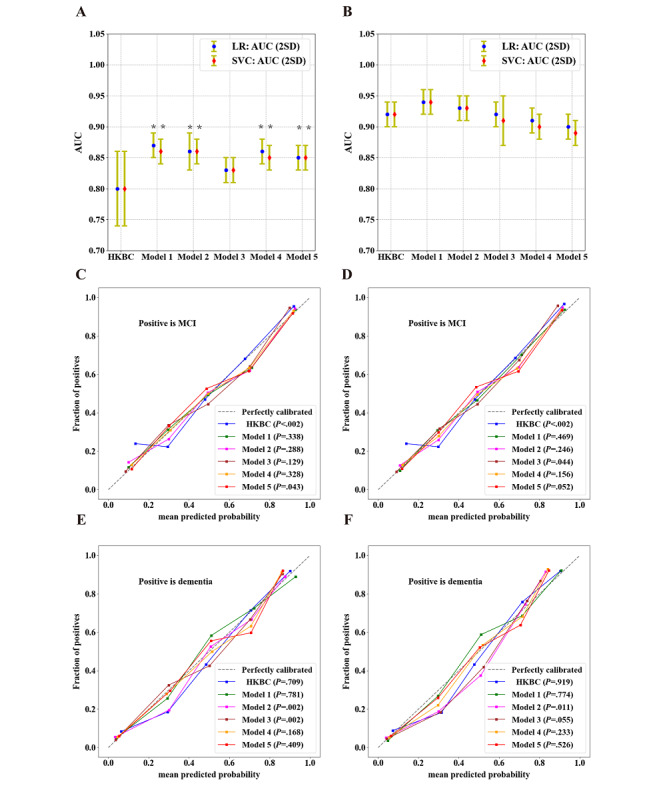
Performance of different models in the CN-NORM cohort. AUC results of different models by different methods of discrimination between (A) MCI and CN groups and between (B) MCI and dementia groups. (C, D) Calibration plots of different models on MCI using LR and SVC, respectively. (E, F) Calibration plots of different models on dementia using LR and SVC, respectively. AUC and calibration plots were drawn based on LR and SVC methods, respectively. A 3-fold cross-validation strategy was followed to calculate the results. The DeLong test for AUC comparison was conducted between different models and the reference (HKBC test) on plots (A) and (B). *P*<.05 indicated a statistically significant difference. Calibration plots (C-F) were evaluated with *χ*^2^ goodness-of-fit tests on predicted probabilities and observed proportions of events, where P≥.05 indicated the goodness of fit. *P<.05. AUC: area under the curve; CN: cognitive normal; HKBC: Hong Kong Brief Cognitive; MCI: mild cognitive impairment; LR: logistic regression; SVC: support vector classification.

The performance of each model was assessed in terms of discrimination and calibration, as shown in Figure 5 and Table S8 in Multimedia Appendix 1. For the MCI and CN groups, compared to the HKBC test, all models, except model 3, had a higher AUC and the DeLong test result was significant for all models (*P*<.05) according to LR and SVC algorithms (Figure 5A). For the MCI and dementia groups, the discrimination ability of all models was also similar to that of the HKBC test score, with all *P*≥.05 (Figure 5B). Model 5 provided the highest sensitivity among all models: up to 0.82 (range 0.72-0.92) and 0.83 (range 0.75-0.91) according to LR and SVC algorithms, respectively. The positive predictive value (PPV) and negative predictive value (NPV) of this model were similar as that of the HKBC test: up to 0.78 (range 0.68-0.88) and 0.73 (range 0.71-0.75), respectively (Table S8 in Multimedia Appendix 1).

Calibration plots were generated and are shown in Figure 5. Compared to the HKBC test, model 5 (LR), and model 3 (SVC), models 1, 2, and 4 exhibited relatively good calibration for discrimination between the MCI and CN groups (*P*≥.05) according to both LR and SVC (Figures 5C and 5D, respectively). All models, except model 2 (LR and SVC) and model 3 (LR), achieved satisfactory calibration for discrimination between the MCI and dementia groups according to both LR and SVC, with all *P*≥.05 (Figure 5E and 5F, respectively).

Model 5 achieved a higher level of discrimination as the HKBC test score in distinguishing between the MCI and CN groups. This model also exhibited a satisfactory goodness of fit in calibration, given the perfectly calibrated results of the MCI and CN groups’ comparison using the SVC algorithm as well as the MCI and dementia groups’ comparison after independent verification using LR and SVC algorithms (*P*≥.05). Overall, model 5 had both high scalability and discrimination ability for MCI and dementia.

### External Validation in the ADNI-3 Cohort

**Figure 6:**
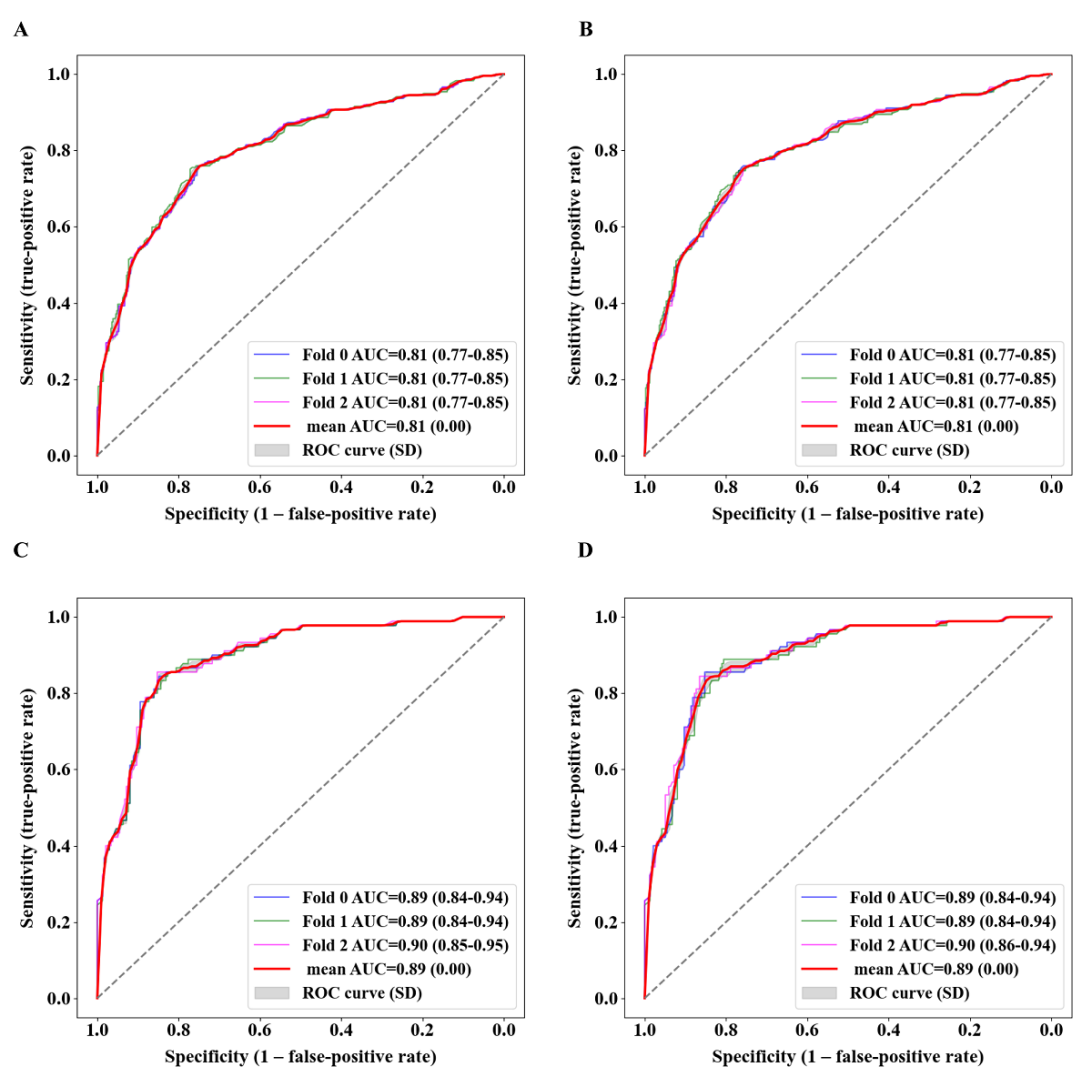
External validation of the CNCB-MCI model (model 5) in the ADNI-3 cohort using different ML methods. (A, B) The AUC plots of model 5 by LR and SVC for discrimination of MCI from CN, respectively. (C, D) The AUC plots of model 5 by LR and SVC for discrimination of dementia from MCI, respectively. A 3-fold cross-validation strategy was followed to calculate the results. AUC plots were drawn based on LR and SVC methods, respectively. CN, cognitive normal controls; MCI, mild cognitive impairment; AUC, Area Under Curve; LR, logistic regression; SVC, Support Vector Classification; ADNI, Alzheimer’s Disease Neuroimaging Initiative.

We further validated model 5 in the ADNI-3 cohort. This panel of cognitive tests yielded similar robust discriminative performance in the ADNI-3 cohort regarding differentiation between the MCI and CN groups, with a mean AUC of up to 0.81 (SD 0) with both LR and SVC algorithms (Figures 6A and 6B, respectively). Regarding differentiation between the MCI and dementia groups, this model also achieved good discriminative ability, with a mean AUC value of up to 0.89 (SD 0) according to both LR and SVC algorithms (Figures 6C and 6D, respectively). The sensitivity, specificity, PPV, and NPV of model 5 regarding differentiation between the MCI and CN groups with LR were up to 0.76 (range 0.76-0.76), 0.76 (range 0.74-0.78), 0.73 (range 0.71-0.75), and 0.78 (range 0.78-0.78), respectively (Table S8 in Multimedia Appendix 1). Therefore, model 5 had good generalizability to the ADNI-3 cohort (Figure 6).

## Discussion

### Principal Findings

Using ML methods, we developed a stable and scalable digital composite neurocognitive test (model 5) based on the CNCB that could distinguish not only between MCI and CN groups but also between MCI and dementia groups. Compared to the HKBC test, this composite neurocognitive test achieved similar discrimination and better calibration abilities in differentiating between MCI and CN groups as well as between MCI and dementia groups. Moreover, the test was more scalable, contained only 2 brief tests, and was more readily accepted by the elderly. It took approximately 5 minutes to complete the test. The test also had good generalizability to the ADNI-3 cohort. Overall, this digital composite neurocognitive test has both high scalability and high stability for the early discrimination of dementia. It could be not only used as a feasible and practical digital biomarker in large-scale cognition screening but also used in intervention studies.

### Comparison With Other Studies

The digital composite neurocognitive test includes 2 simple and short tests, namely the Hopkins Verbal Learning Test-5 minutes Recall and the Trail Making Test-B, which evaluate memory and executive function, respectively. Both tests in this model were also common dominants identified as candidate variables according to the 4 algorithms used. The Hopkins Verbal Learning Test is a brief, multicomponent word list–learning task that is commonly used to assess verbal learning and memory [21]. It showed the best performance in feature selection, with the highest mean AUC and *F*-score. To reduce the effect of learning, there are 6 alternate versions of the Hopkins Verbal Learning Test, and some of them have shown good intertest reliability in Chinese populations [22]. Therefore, the Hopkins Verbal Learning Test can be frequently used to evaluate cognitive change. The Trail Making Test consists of 2 parts (A and B) and is one of the most frequently used measures to distinguish subjects with cognitive impairment from CN subjects in clinical neuropsychology [23]. Parts A and B are widely used to assess the cognitive processing speed and executive function, respectively [23]. To minimize the impact of linguistic and cultural diversity, many variants of the Trail Making Test have been developed; the version in the CNCB is the Color Trail Test.

Although cognition is multifaceted and MCI can affect many different cognitive domains [24], assessing cognitive function in all domains with a short test is not feasible; thus, instruments must strike a proper balance between the duration and depth of testing to maximize their utility [9]. A consensus of clinical and research experts focused on MCI and AD indicated that a useful cognitive tool should encompass multiple cognitive domains and, at a minimum, assess memory and executive function [9]. The memory and executive function cognitive domains have been extensively validated as sensitive measures of early cognitive decline in AD [25,26]. In this study, cognitive function in 6 cognitive domains suggested that memory and executive function are the 2 cognitive domains most severely impaired in MCI. Guo et al [27] developed a brief cognitive test for detecting MCI only covering the memory and executive function domains [27]. However, our digital composite neurocognitive test contains just 2 brief tests, which is more time efficient and is more readily accepted by the elderly.

This brief composite neurocognitive test is digital, which entails the following advantages. First, it is easy to operate and no specialized training is needed. The test can be administered with the help of caregivers or nurses or can even be self-administered at home following the manual. Moreover, a digital platform with standardized operation can provide consistent analysis and interpretation, and automated scoring. This computerized test is more suitable for populations with low levels of education, large sample sizes, and establishment of test norms [28]. Therefore, it could serve as a suitable tool for large-scale cognitive screening in community and primary care settings.

### Strengths of the Study

This study has a few strengths. The candidate variables and models were based on comprehensive neuropsychological assessments from the CNCB [11], which contains 31 primary variables and 24 cognitive subsets covering 6 cognitive domains. All these subsets are culturally appropriate and have been validated in Chinese individuals. The models developed from the CNCB are more reliable and suitable for populations with low levels of education. Second, in this study, to ensure that the results were more reliable and credible, multiple different ML algorithms were used for feature selection and assessment. In the feature selection stage, we adopted the *F* test, logit regression (and corresponding *P* value), the AUC according to LR, and backward stepwise elimination. The optimal results of these 4 methods all indicated the 2 variables included in model 5. We also adopted a stratified cross-validation method based on sampling. This method ensures that the training set is consistent with the overall distribution in data labels and features, ensuring the mean of the 3-fold results is closer to the true value of the population. We independently validated the ROC and AUC results of LR based on the maximum likelihood estimation and the SVC algorithm based on structural risk minimization. The results showed no significant differences between the 2 algorithms. Third, this study was a nationwide multicenter study in China, and the MCI group contained different subtypes of MCI, including amnestic and nonamnestic MCI; the dementia group included AD, frontotemporal dementia, and dementia with Lewy bodies. Models derived from this heterogeneous population will be more stable when used in other populations. Fourth, reports of excellent sensitivity and specificity for a given instrument must consider the possibility that performance is inflated by high rates of dementia. In this study, we assessed the diagnostic performance of composite tests in separate groups of patients with MCI and dementia.

### Weaknesses of the Study

This study has a few limitations. First, the diagnosis of MCI or dementia was based on DSM-5 criteria. In this study, confirmed biomarkers, such as the CSF or PET imaging, were lacking. Confirmation with biomarkers could improve the credibility of results. Second, the cognitive tests used in the ADNI-3 cohort were slightly different from those used in the CN-NORM cohort, which may cause information bias. However, the core of these cognitive tests was the same, and they were implemented in similar ways. Third, this study was based on a cross-sectional design. Further longitudinal studies with larger sample sizes may explore the ability of this brief composite neurocognitive test to monitor the conversion from normal cognition to MCI and dementia. Fourth, although this study was a multicenter study conducted across China, all participants were subjects who were willing to undergo cognitive assessment rather than individuals randomly recruited from the community. Thus, this selection bias may limit the generalizability of our findings. Fifth, although we adjusted for age, gender, and education when calculated cognitive function, we could not rule out the possibility of confounding factors, such as sleep-related issues, anxiety, and depression, that may affect cognitive function. Six, the *z*-score was converted based on the current normal group data rather than all normal subject in China and SDs may be smaller than they actually were. In addition, as distributions of the most of test scores were not normal, *z*-score conversion may also introduce some bias. However, it is one of the commonly used methods in the neurocognitive field [17-19], and the results of this method are more understandable and acceptable to professionals in the neurocognitive field.

### Conclusion

We developed a stable and scalable digital composite neurocognitive test based on ML that can differentiate not only MCI from normal cognition but also MCI from dementia. This digital test consists of the Hopkins Verbal Learning Test-5 minutes Recall and the Trail Making Test-B and is time efficient and easily administered. The test represents a feasible and practical digital biomarker for use in large-scale cognitive screening and might be useful in intervention studies.

## Appendix

Multimedia Appendix 1 Supplementary materials.

## Abbreviations

AD: Alzheimer’s disease
ADAS-Cog: Alzheimer’s Disease Assessment Scale-Cognitive
ADNI: Alzheimer’s Disease Neuroimaging Initiative
AUC: area under the curve
CN: cognitively normal
CNCB: Chinese Neuropsychological Consensus Battery
CN-NORM: Chinese Neuropsychological Normative Project
CSF: cerebrospinal fluid
DSM-5: *Diagnostic and Statistical Manual of Mental Disorders, Fifth Edition*
GDS: Geriatric Depressive Scale
HKBC: Hong Kong Brief Cognitive
H-L: Hosmer-Lemeshow
LR: logistic regression
MCI: mild cognitive impairment
ML: machine learning
NCD: neurocognitive disorder
NPV: negative predictive value
PET: positron emission tomography
PPV: positive predictive value
ROC: Receiver-operating characteristic
SVC: support vector classification

## Data Availability

The data sets generated and analyzed during the study are available from the corresponding author upon reasonable request.
