# Supplementary material for: A Stable and Scalable Digital Composite Neurocognitive Test for Early Dementia Screening Based on Machine Learning: Model Development and Validation Study
Source: J Med Internet Res. 2023 Dec 1;25:e49147. doi: 10.2196/49147 (PMC10724812; doi:10.2196/49147)
Supplement: Multimedia Appendix 1 [file jmir_v25i1e49147_app1.docx]

Supplementary materials

**Methods**

**Neuropsychological assessment**

Testing occur in a quiet room with as few distractions as possible. Because the many ways in which the examiner can influence testing, the same rater administered all tests for a study participant. Before the testing, the rater explained to the participant the purpose of the testing, what the tests would be like, how long testing would take, and what the day’s schedule would be. In order to avoid interference with each other during experimental process, cognitive tests related memory should not be performed next to each other, such as Hopkins verbal learning test and logical memory subtests. During the delayed recall of memory tests, other tests were performed. At the end of delayed recall time, the electronic assessment system would remind assessors.

Specifically, attention was assessed using the digit span-forward subtest of the Wechsler Adult Intelligence Scale-Revised for China (WAIS-RC)^1^ and Trail Making Test and Trail Making Test-A^2^. Memory was assessed using logical memory subtests from the Wechsler Memory Scale-Revised for China (WMS-RC)^3^, Hopkins verbal learning test^4^ and the Brief visuospatial memory test^5^. We examined executive function using the Trail making test-B^2^, Digit symbol substitution test, Stroop^6^ and Digit span-backward subtest of the WAIS-RC^1^. Language ability was evaluated using the Verbal Fluency-Animal Naming^7^ and Boston Naming tests^8^. Visuospatial ability was examined using the Clock Drawing Test (4 points), the Judgment of Line Orientation Test^9^ and Visual Object and Space Perception-Silhouettes^10^. Social cognition was assessed by Eye Emotional Recognition Task.

All tests were given in the same order with adherence to time limit and standardized instructions. The recommended sequence of cognitive assessments was as follows: (1) Clock Drawing Test; (2) Verbal Fluency-Animal Naming; (4) HKBC; (5) Brief Visual Memory Test; (6) Trail Making Test; (7) Trail Making Test-A; (8) Trail Making Test-B; (9) Digit Span-Forward; (10) Digit Span-Backward; (11) Brief Visual Memory Test-30 minutes Recall; (12) Digit Symbol Substitute Test; (13) Hopkins Verbal Learning Test; (14) Stroop Word Test; (15) Stroop Color test; (16) Stroop Color-Word Test; (17) Hopkins Verbal Learning Test-5 minutes recall; (18) Judgment of Line Orientation Test; (19) Hopkins Verbal Learning Test -20 minutes Recall; (20) Silhouettes; (21) Logic Memory Test; (22) Eye Emotional Recognition Task-Gender; (23) Eye Emotional Recognition Task-Mood; (24) Boston Naming Test; (25) Logic Memory Test-30 minutes Recall.

**Assessment of Depressive Symptoms**

In the CN-NORM study, depressive symptoms were evaluated using the Chinese version of the 30-item Geriatric Depressive Scale (GDS)^11,12^, which is commonly used to assess depression in older adults. Scores equal to or higher than 10 suggest the presence of depressive symptoms (DS). In the ADNI study, the 15-item GDS was used to identify symptoms of depression in elderly individuals. Total scores of 6–15 were considered to indicate depression.

Table S1 Detail of the neuropsychological battery of CNCB and cognitive tests in ADNI cohort

| **Domain** | **Cognitive tests** | **Task** | **Variables** | **Scoring** | **Range**  **(min - max)** |
| --- | --- | --- | --- | --- | --- |
| **CN-NORM cohort** | | | | |  |
| Attention | Digit Span-Forward | Repeat a sequence of digits in forward. | Digit Span-Forward | The number and length of groups of numbers in correct order. | 0-14 |
|  |  |  | Digit Span-Forward Length |  | 0-9 |
|  | Trail Making Test | Connect 25 numbered back circles in ascending numerical order. | Trail Making Test | Time (seconds) to complete the task. | 0-300 |
|  | Trail Making Test-A | Connect 25 numbered colored circles in ascending numerical order. | Trail Making Test-A | Time (seconds) to complete the task. | 0-300 |
| Memory | Hopkins Verbal Learning Test | Listen to 12 words 3 times and recall them afterwards. | Hopkins Verbal Learning Test-1 | Number of correct words subjects recalled. | 0-12 |
|  |  |  | Hopkins Verbal Learning Test-2 |  |  |
|  |  |  | Hopkins Verbal Learning Test-3 |  |  |
|  | Hopkins Verbal Learning Test-5 minutes Recall | Listen to 12 words 3 times and recall them after 5 minutes. | Hopkins Verbal Learning Test-5 minutes Recall | Number of correct words subjects recalled. | 0-12 |
|  | Hopkins verbal learning test -20 minutes recall | Listen to 12 words 3 times and recall them after 20 minutes. | Hopkins Verbal Learning Test-20 minutes Recall | Number of correct words subjects recalled. | 0-12 |
|  | Brief Visual Memory Test | The subject was shown a set of geometric shapes and drawn them as accurately as possible for three times. | Brief Visual Memory Test-1 | One point for each correct shape and position for every time. | 0-12 |
|  |  |  | Brief Visual Memory Test-2 |  |  |
|  |  |  | Brief Visual Memory Test-3 |  |  |
|  | Brief Visual Memory test-30 minutes Recall | The subject was shown a set of geometric shapes and drawn them as accurately as possible after 30 minutes. | Brief Visual Memory Test-30 minutes Recall | One point for each correct shape and position, respectively. | 0-12 |
|  | Logic Memory Test | Listen to 2 stories and recall it as accurately as possible, respectively. | Logic Memory-1 | Number of correct words in the stories that subjects could recall. | 0-20 |
|  |  |  | Logic Memory-2 |  |  |
|  | Logic Memory Test-30 minutes Recall | Listen to 2 stories and recall it after 30 minutes as accurately as possible, respectively. | Logic Memory-1-30 minutes recall | Number of correct words in the stories that subject could recall after 30 min. | 0-30 |
|  |  |  | Logic Memory-2-30 minutes Recall |  |  |
| Executive Function | Trail Making Test-B^a^ | Connect 25 numbered circles while alternating colors in ascending numerical order. | Trail Making Test-B | Time (seconds) to complete the task. | 0-300 |
|  | Digit Span-Backward | Repeat a sequence of digits in backward. | Digit Span-Backward | The number of groups of numbers in correct order. | 0-14 |
|  |  |  | Digit Span-Backward Length | The max length of groups of numbers in correct order. | 0-8 |
|  | Digit Symbol Substitute Test | Select the correct number of symbols within 90 seconds. | Digit Symbol Substitute Test | Number of correct symbols subjects selected. | ≥ 0 |
|  | Stroop Word Test | Select the same words as the ones given. | Stroop Word Test | Number of correct words subjects selected. | 0-110 |
|  | Stroop Color Test | Select the colors as the words given. | Stroop Color Test | Number of correct words subjects selected. | 0-110 |
|  | Stroop Color-word Test | Select the colors as the colors words given. | Stroop Color-Word test | Number of correct words subjects selected. | 0-110 |
| Language | Verbal Fluency-Animal Naming | The subject has to name as many animals as possible in 60 seconds. | Animal Naming | Number of correct animal names. | ≥ 0 |
|  | Boston Naming Test | The subject has to name 30 images. | Boston Naming Test | Number of correctly recognised items. | 0-30 |
| Visuospatial function | Clock Drawing Test | Draw a circular clock with all numbers, then set the time to 8:20. | Clock Drawing Test | Criteria: contour, numbers, position of the number, hands. | 0-4 |
|  | Judgment of Line Orientation Test | Selects line segments in the same direction of the given ones. | Line Orientation Test | The number of groups of correct line segment selected. | 0-30 |
|  | Silhouettes | Name 15 silhouettes. | Silhouettes | Number of correctly recognised items. | 0-15 |
| Social Cognition | Eye Emotional Recognition Task | Participants were shown 34 images of the eye area and then judged the mood and gender of the person in the image. | Eye Emotional Recognition Task-Mood | Number of correctly recognised items. | 0-34 |
|  |  |  | Eye Emotional Recognition Task-Gender | Number of correctly recognised items. | 0-34 |
| **ADNI cohort** | | | | | |
| Memory | ADAS-Cog word recall | Participants were shown 10 words 3 times and recall these words later. | ADAS-Cog word recall | Number of correct words subjects recalled. | 0-10 |
| Executive Function | Trail Making Test-B^b^ | Connect the circles while alternating between numbers and letters in an ascending order (e.g., A to 1; 1 to B; B to 2; 2 to C) | Trail Making Test-B | Time (seconds) to complete the task. | 0-300 |

a, the subject is instructed to connect numbered circles while alternating colors; b, the subject is instructed to connect the letters and numbers alternatively. Abbreviations: CNCB, Chinese Neuropsychological Consensus Battery; CN-NORM, Chinese Neuropsychological Normative Project; ADNI, Alzheimer’s Disease Neuroimaging Initiative.

Table S2 Comparison of continuous variables data distribution among CN, MCI and dementia group in CN-NORM cohort

| **Demographic characteristic variables** | |
| --- | --- |
| 1 Age | 2 GDS |
| 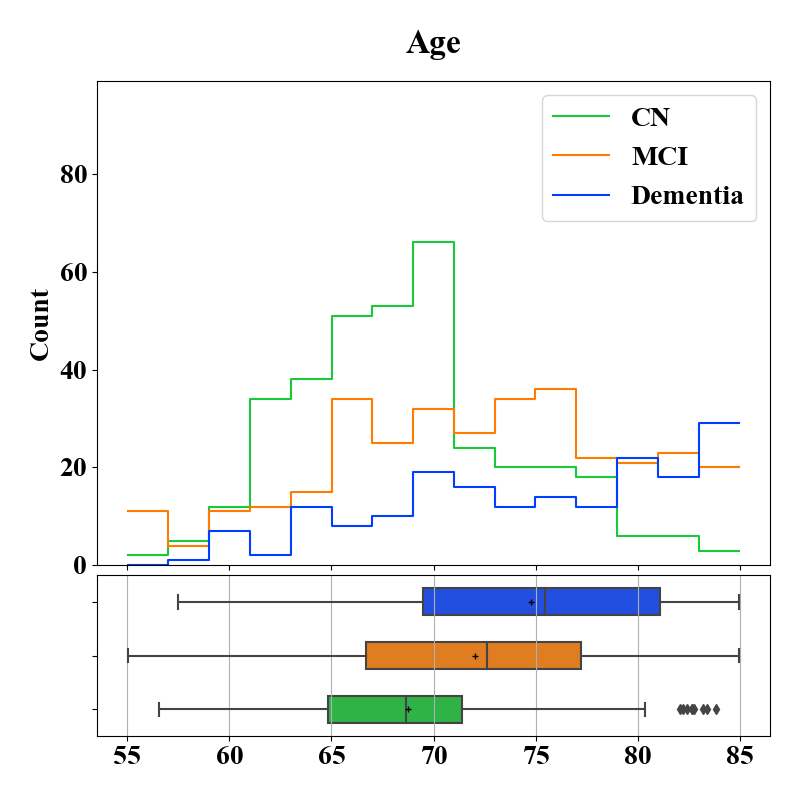 | 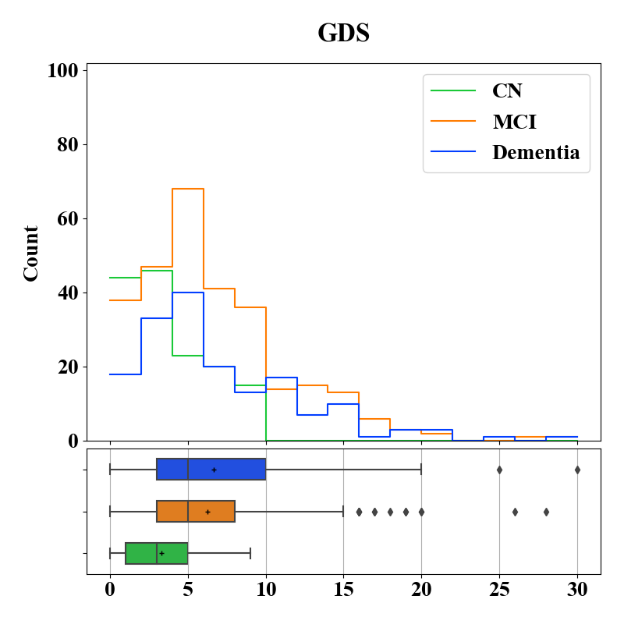 |
| **Raw neuropsychological scores in CNCB** | |
| 1 Digit Span-Forward | 2 Digit Span-Forward Length |
| 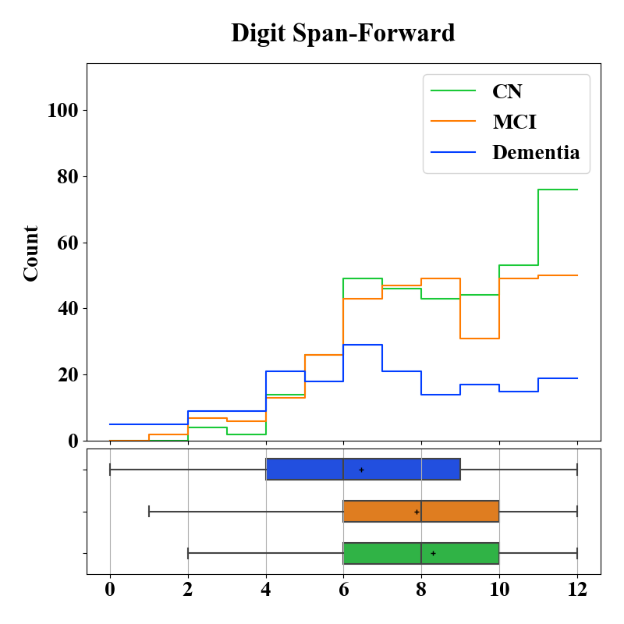 | 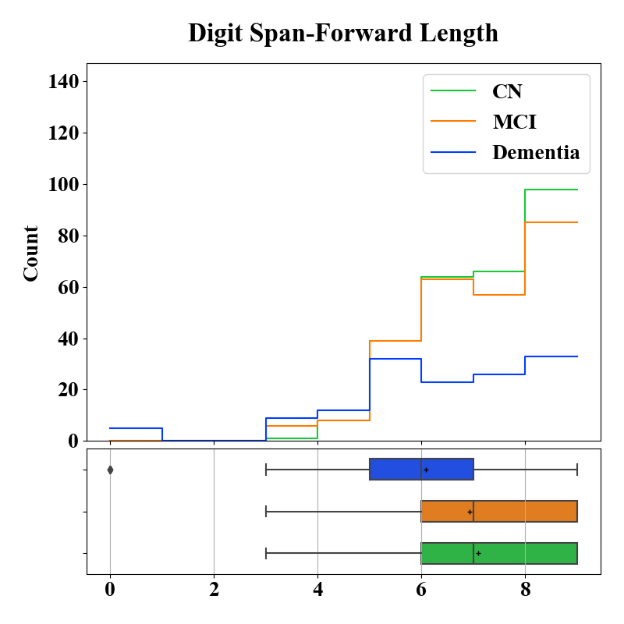 |
| 3 Trail Making Test | 4 Trail Making Test-A |
| 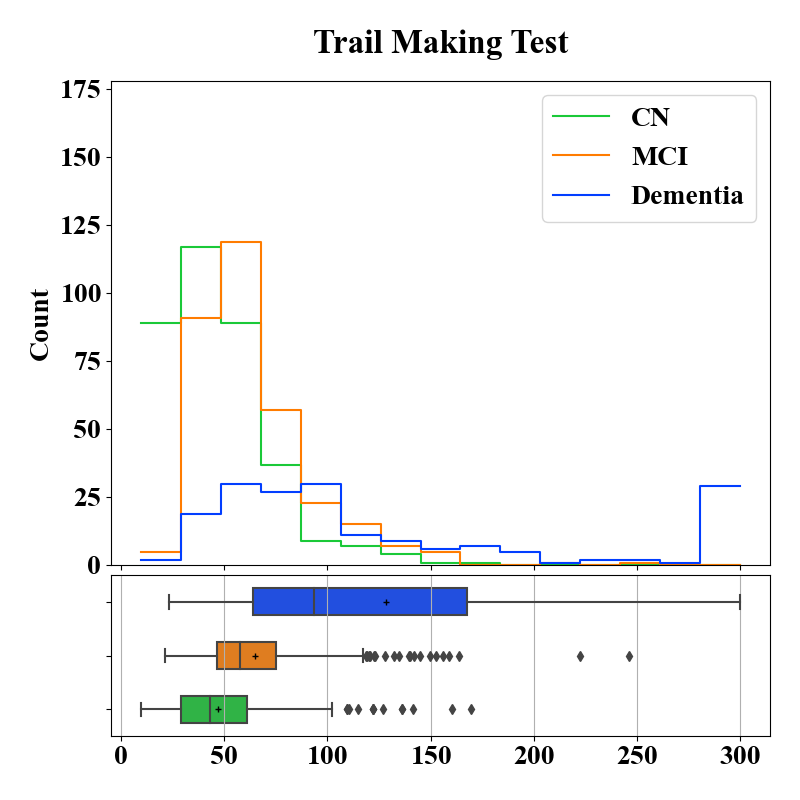 | 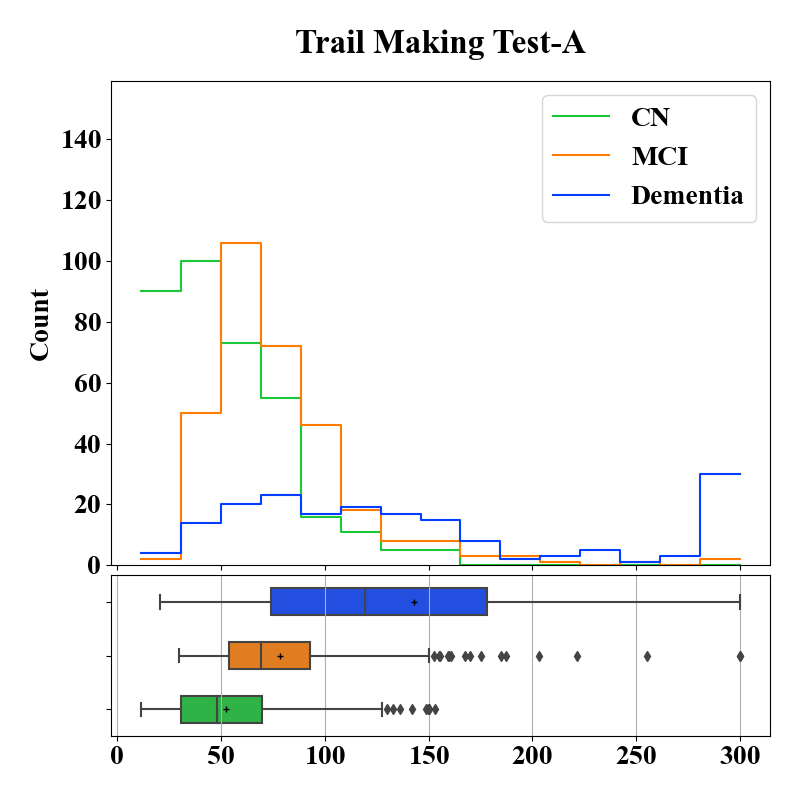 |
| 5 Hopkins Verbal Learning Test-1 | 6 Hopkins Verbal Learning Test-2 |
| 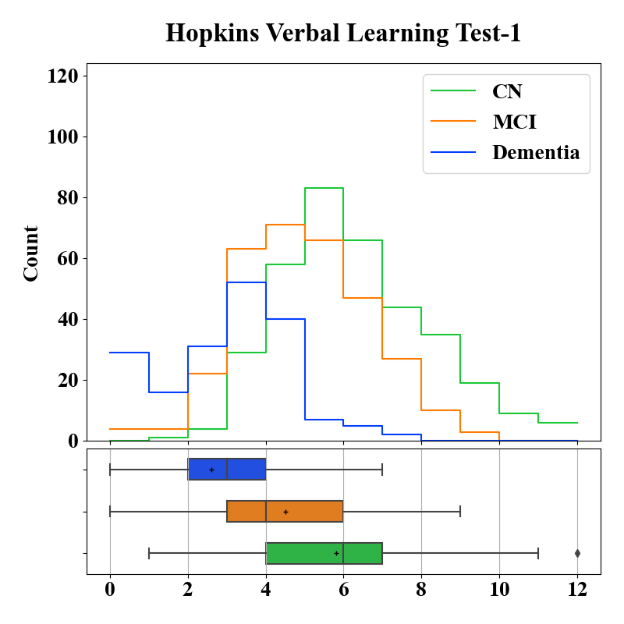 | 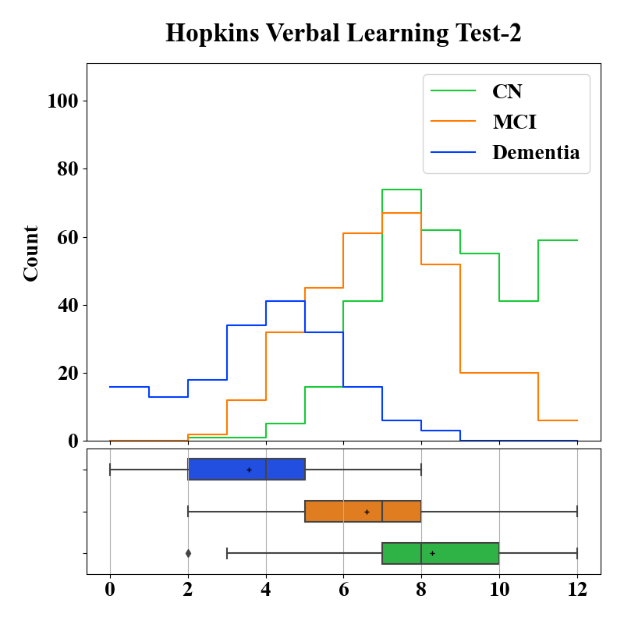 |
| 7 Hopkins Verbal Learning Test-3 | 8 Hopkins Verbal Learning Test-20 minutes Recall |
| 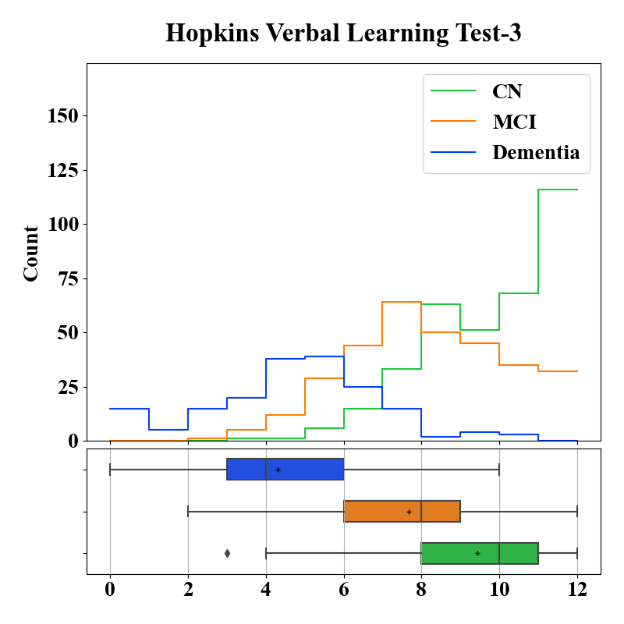 | 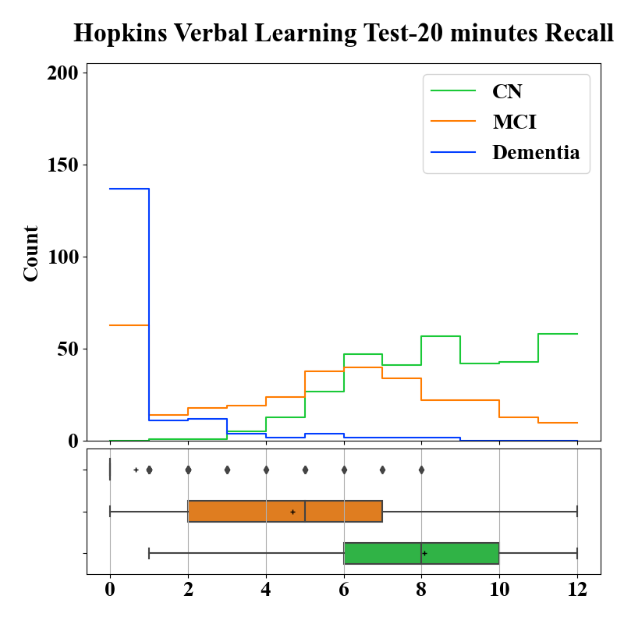 |
| 9 Brief Visual Memory Test-1 | 10 Brief Visual Memory Test-2 |
| 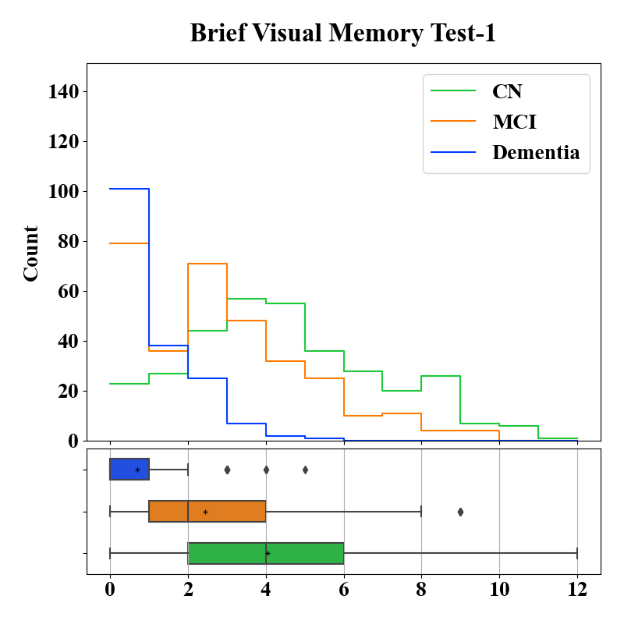 | 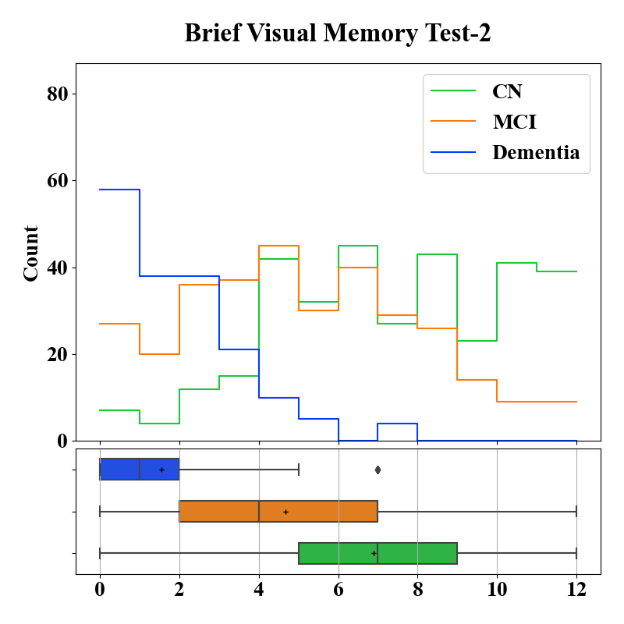 |
| 11 Brief Visual Memory Test-3 | 12 Brief Visual Memory Test-30 minutes Recall |
| 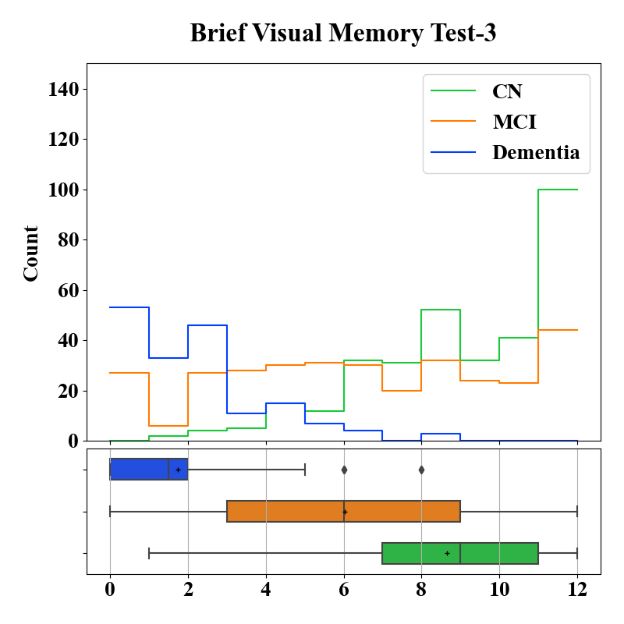 | 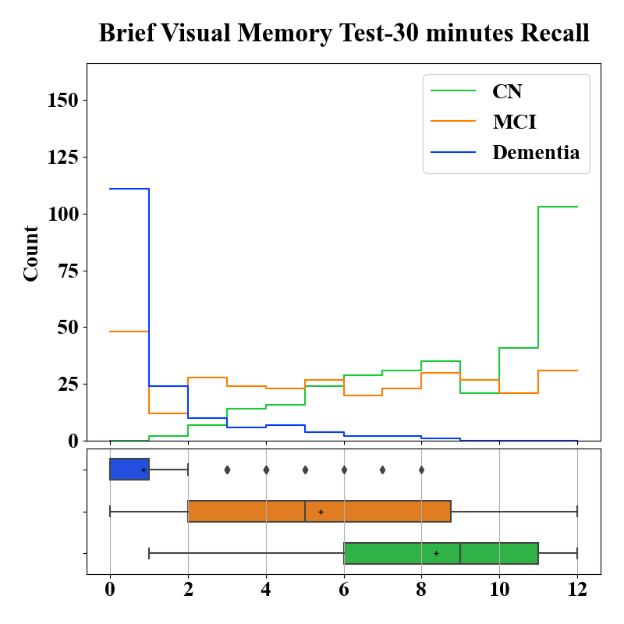 |
| 13 Logic Memory-1 | 14 Logic Memory-2 |
| 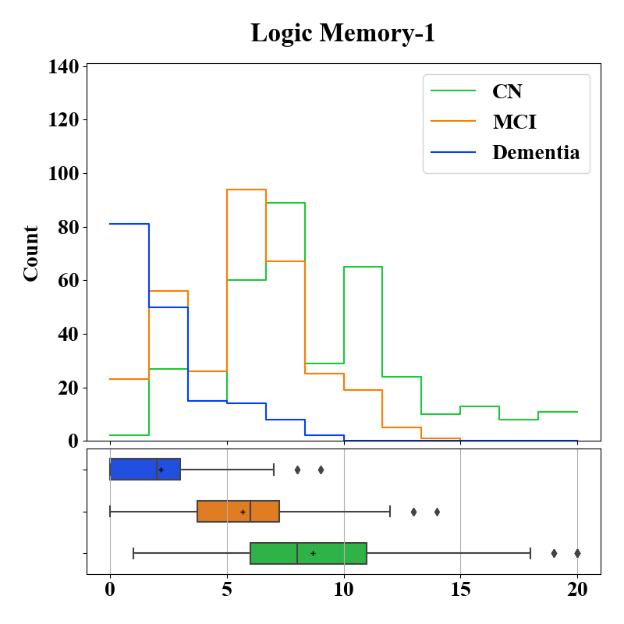 | 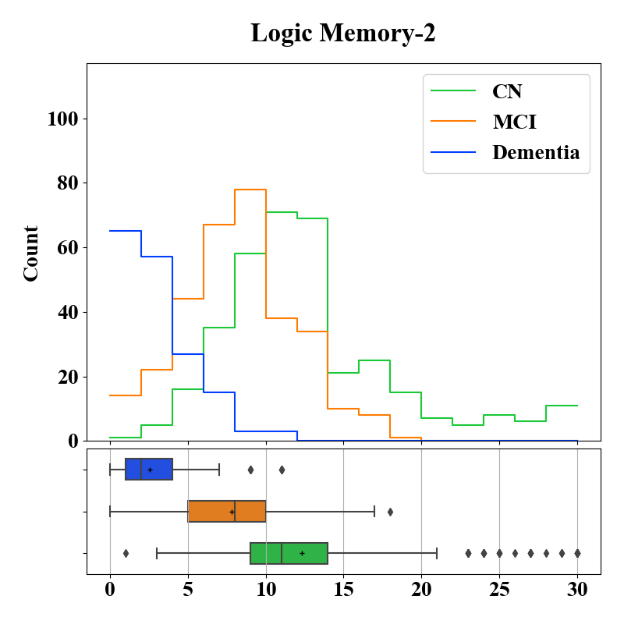 |
| 15 Logic Memory-30 minutes Recall-1 | 16 Logic Memory-30 minutes Recall-2 |
| 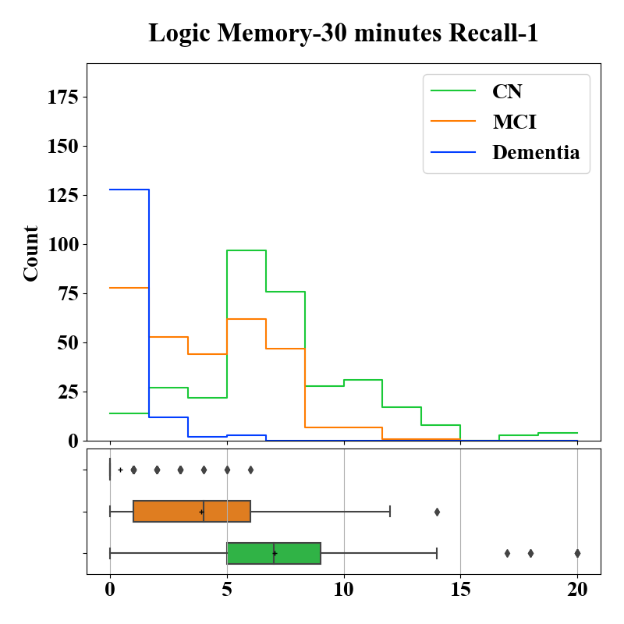 | 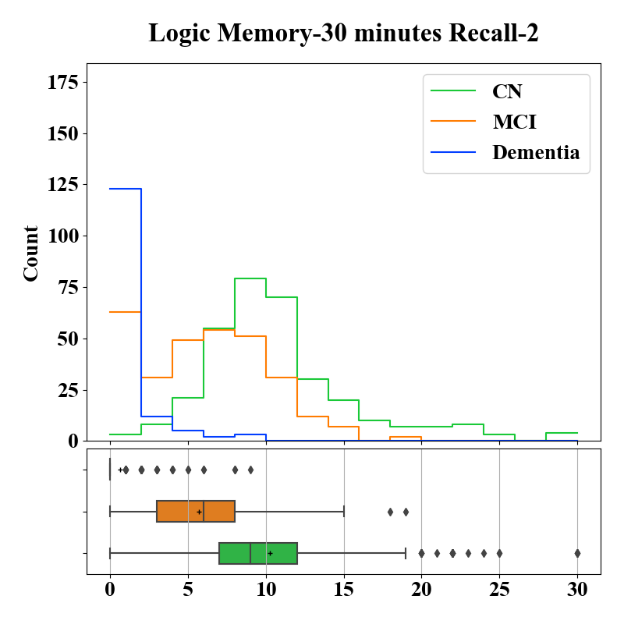 |
| 17 Digit Span-Backward | 18 Digit Span-Backward Length |
| 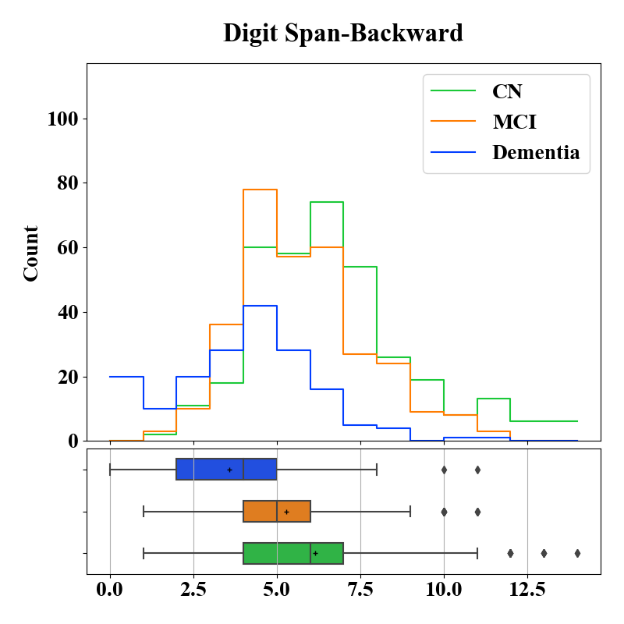 | 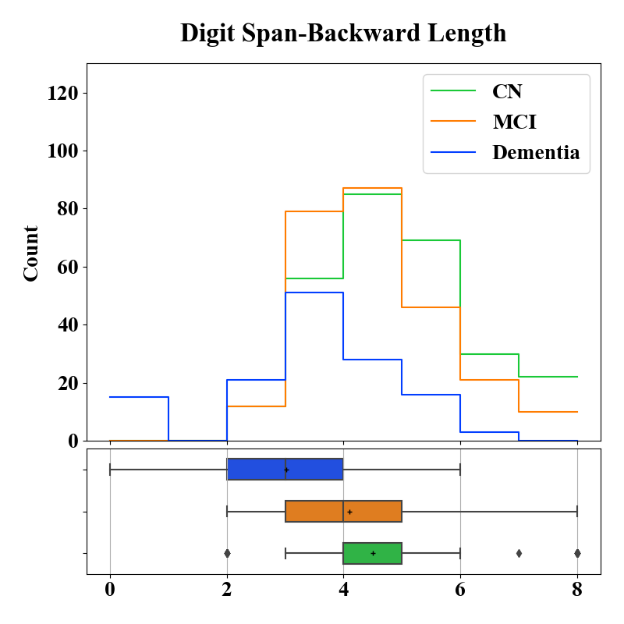 |
| 19 Digit Symbol Substitute Test | 20 Stroop Word Test |
| 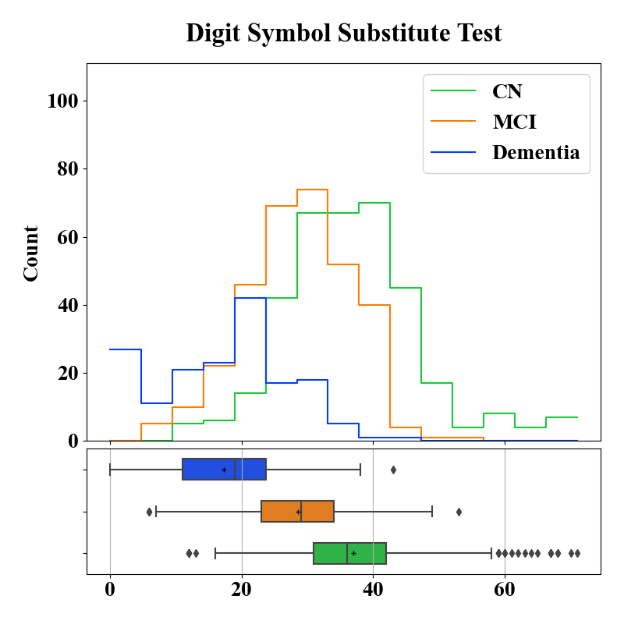 | 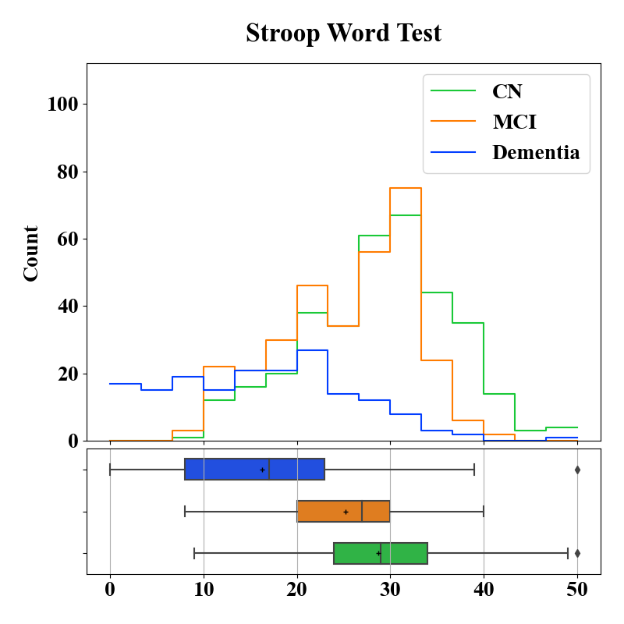 |
| 21 Stroop Color Test | 22 Stroop Color-Word Test |
| 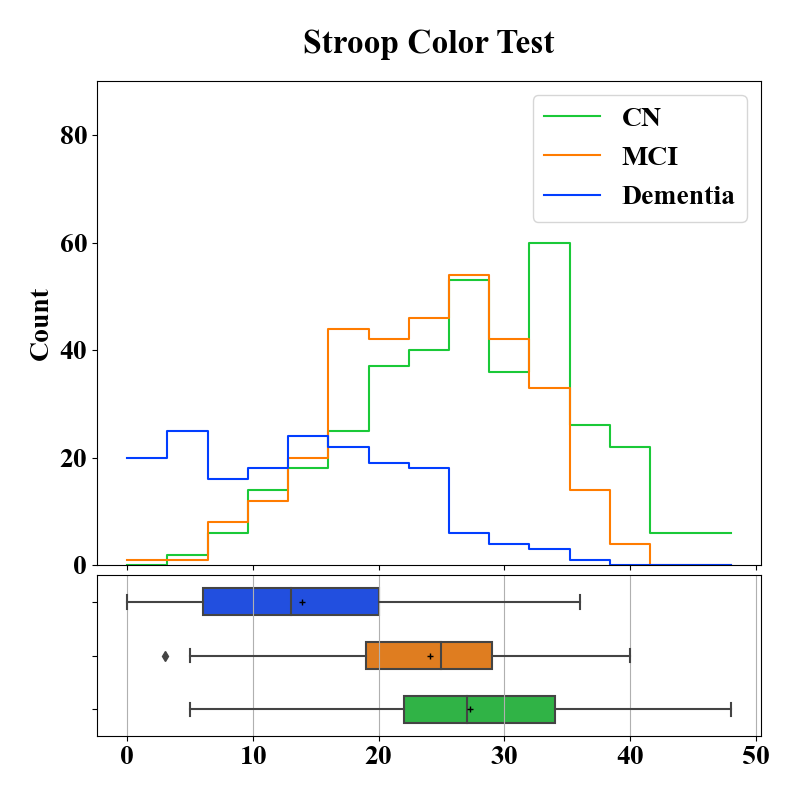 | 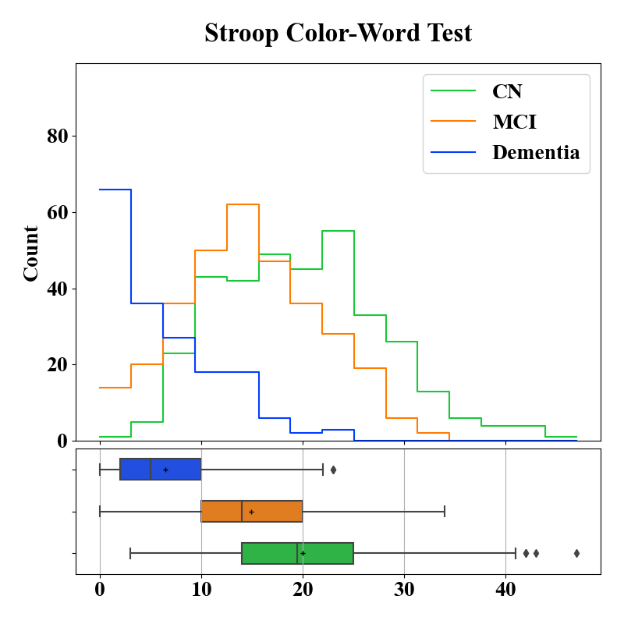 |
| 23 Animal Naming | 24 Boston Naming Test |
| 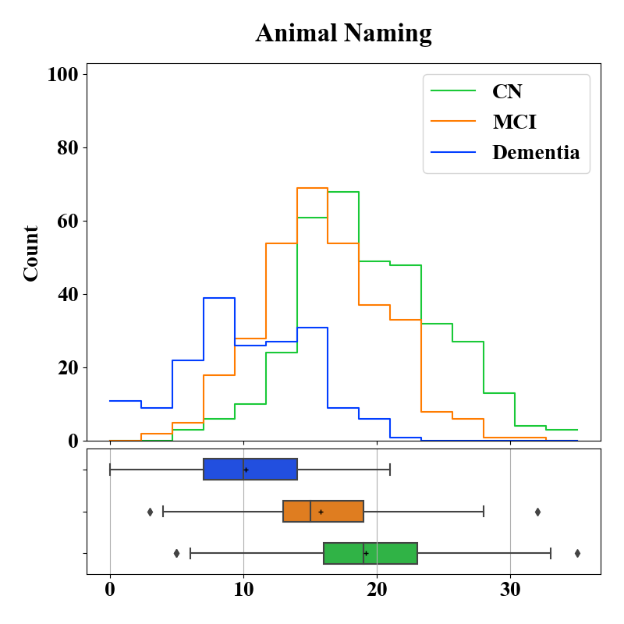 | 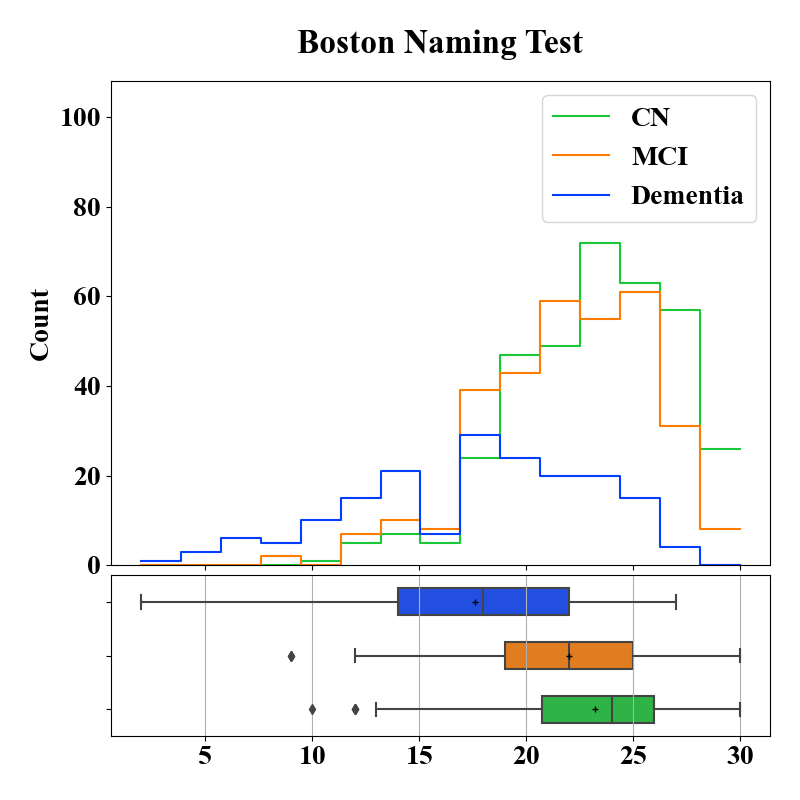 |
| 25 Clock Drawing Test | 26 Line Orientation Test |
| 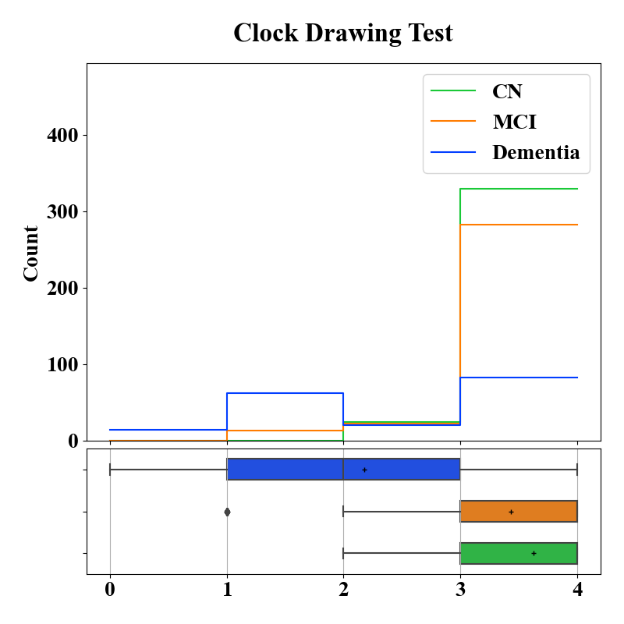 | 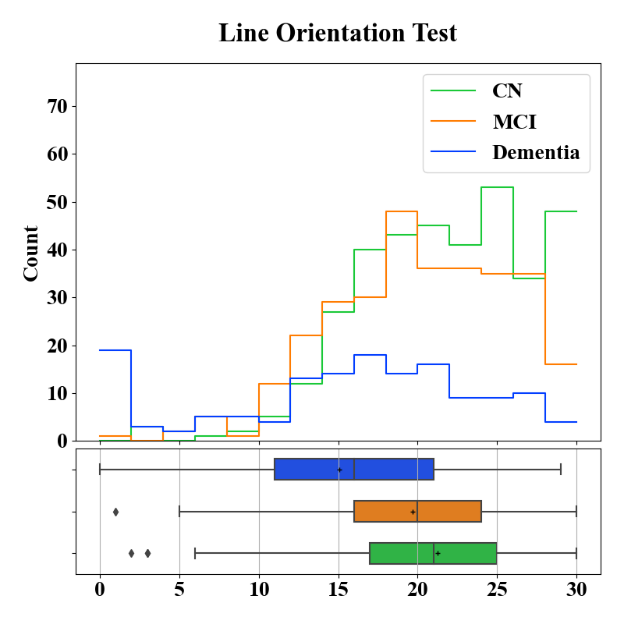 |
| 27 Silhouettes | 28 Eye Emotional Recognition Task-Mood |
| 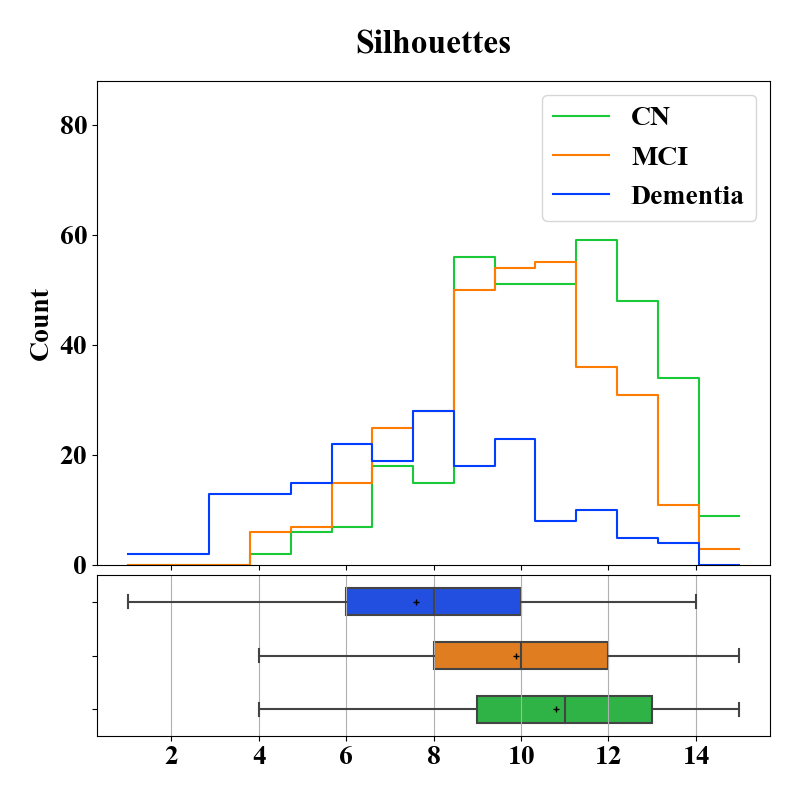 | 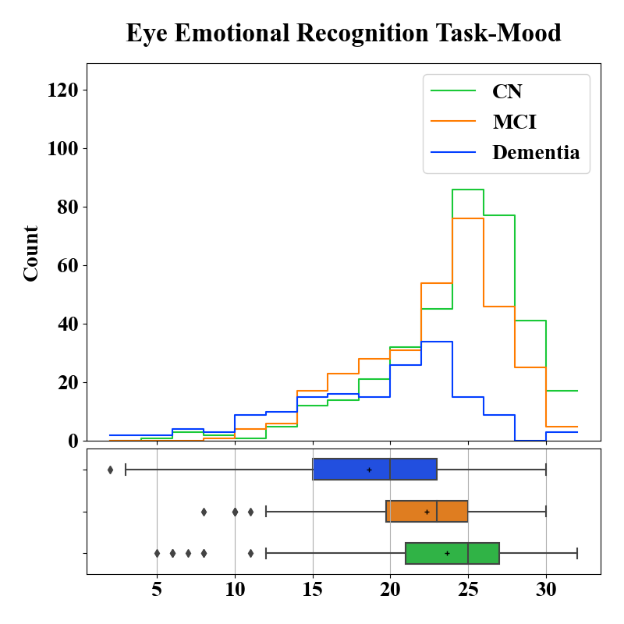 |
| 29 Eye Emotional Recognition Task-Gender |  |
| 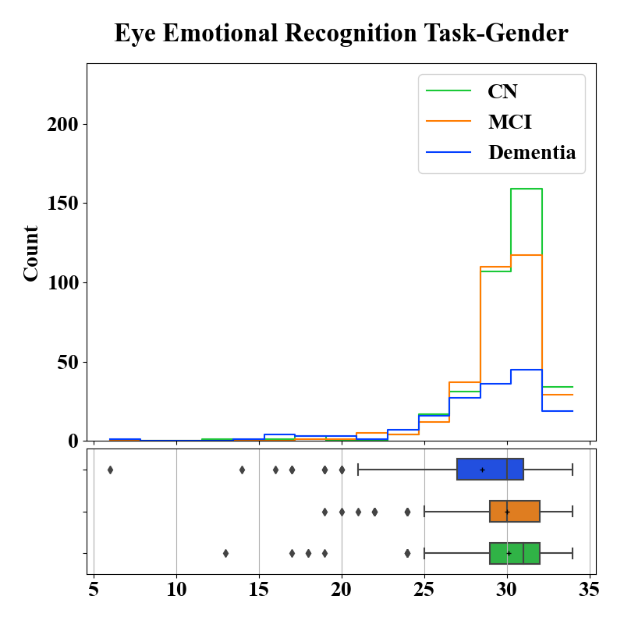 |  |

Table S3 Comparisons of raw neuropsychological scores between CN, MCI and dementia groups in both CN-NORM and ADNI cohorts

| Instruments and domains | CN  Mean (SD) | MCI  Mean (SD) | Dementia  Mean (SD) | *H^c^* | *P* values | *P* values | |
| --- | --- | --- | --- | --- | --- | --- | --- |
|  |  |  |  |  |  | CN vs. MCI | MCI vs Dementia |
| **CN-NORM cohort** |  |  |  |  |  |  |  |
| **Global cognitive function** |  |  |  |  |  |  |  |
| HKBC | 26.92 (1.83) | 23.10 (3.97) | 14.34 (4.58) | 414.91 | <.001 | <.001 | <.001 |
| **Attention** |  |  |  |  |  |  |  |
| Digit Span-Forward | 8.30 (2.42) | 7.88 (2.50) | 6.45 (2.96) | 47.81 | <.001 | .049 | <.001 |
| Digit Span-Forward Length | 7.07 (1.40) | 6.94 (1.49) | 6.06 (1.89) | 22.83 | <.001 | .292 | <.001 |
| Trail Making Test | 46.79 (26.45) | 65.13 (29.32) | 127.72 (86.03) | 228.42 | <.001 | <.001 | <.001 |
| Trail Making Test-A | 52.54 (29.83) | 78.46 (37.37) | 142.77 (85.13) | 245.63 | <.001 | <.001 | <.001 |
| **Memory** |  |  |  |  |  |  |  |
| Hopkins Verbal Learning Test-1 | 5.81 (1.92) | 4.49 (1.66) | 2.61 (1.6) | 272.96 | <.001 | <.001 | <.001 |
| Hopkins Verbal Learning Test-2 | 8.28 (1.97) | 6.61 (1.88) | 3.58 (1.86) | 376.56 | <.001 | <.001 | <.001 |
| Hopkins Verbal Learning Test-3 | 9.45 (1.89) | 7.69 (2.03) | 4.3 (2.16) | 382.77 | <.001 | <.001 | <.001 |
| Hopkins Verbal Learning Test-5 minutes Recall | 8.73 (2.13) | 5.41 (3.21) | 0.98 (1.71) | 438.46 | <.001 | <.001 | <.001 |
| Hopkins Verbal Learning Test-20 minutes Recall | 8.02 (2.32) | 4.70 (3.28) | 0.63 (1.56) | 419.55 | <.001 | <.001 | <.001 |
| Brief Visual Memory Test-1 | 4.05 (2.39) | 2.43 (2.12) | 0.66 (0.98) | 247.10 | <.001 | <.001 | <.001 |
| Brief Visual Memory Test-2 | 6.9 (2.86) | 4.66 (2.89) | 1.52 (1.55) | 304.97 | <.001 | <.001 | <.001 |
| Brief Visual Memory Test-3 | 8.68 (2.49) | 6.03 (3.50) | 1.73 (1.71) | 353.26 | <.001 | <.001 | <.001 |
| Brief Visual Memory Test-30 minutes Recall | 8.4 (2.84) | 5.36 (3.71) | 0.77 (1.56) | 359.54 | <.001 | <.001 | <.001 |
| Logic Memory-1 | 8.66 (3.96) | 5.68 (2.73) | 2.16 (2.05) | 329.24 | <.001 | <.001 | <.001 |
| Logic Memory-2 | 12.28 (5.74) | 7.84 (3.59) | 2.52 (2.37) | 388.22 | <.001 | <.001 | <.001 |
| Logic Memory-30 minutes Recall-1 | 7.39 (3.77) | 3.90 (2.87) | 0.34 (1) | 350.94 | <.001 | <.001 | <.001 |
| Logic Memory-30 minutes Recall-2 | 10.57 (5.23) | 5.72 (3.96) | 0.49 (1.48) | 366.07 | <.001 | <.001 | <.001 |
| **Executive Function** |  |  |  |  |  |  |  |
| Trail Making Test-B^a^ | 106.44 (56.2) | 171.56 (59.01) | 241.67 (70.22) | 325.13 | <.001 | <.001 | <.001 |
| Digit Span-Backward | 6.15 (2.37) | 5.27 (1.90) | 3.60 (2.05) | 135.10 | <.001 | <.001 | <.001 |
| Digit Span-Backward Length | 4.38 (1.28) | 4.08 (1.13) | 3.02 (1.22) | 80.13 | <.001 | 0.004 | <.001 |
| Digit Symbol Substitute Test | 36.92 (10.33) | 28.63 (8.03) | 17.51 (9.19) | 312.02 | <.001 | <.001 | <.001 |
| Stroop Word Test | 28.74 (7.74) | 25.23 (7.02) | 16.31 (9.27) | 184.83 | <.001 | <.001 | <.001 |
| Stroop Color Test | 27.24 (8.60) | 24.13 (7.26) | 13.85 (8.33) | 206.91 | <.001 | <.001 | <.001 |
| Stroop Color-Word Test | 19.98 (7.95) | 14.88 (6.79) | 6.39 (5.32) | 288.42 | <.001 | <.001 | <.001 |
| **Language** |  |  |  |  |  |  |  |
| Animal Naming | 23.22 (3.93) | 22.03 (3.96) | 17.63 (5.34) | 268.76 | <.001 | <.001 | <.001 |
| Boston Naming Test | 19.17 (5.21) | 15.74 (4.60) | 10.15 (4.62) | 135.50 | <.001 | .001 | <.001 |
| **Visuospatial function** |  |  |  |  |  |  |  |
| Clock Drawing Test | 3.63 (0.61) | 3.44 (0.80) | 2.17 (1.32) | 178.21 | <.001 | 0.007 | <.001 |
| Line Orientation Test | 21.23 (5.27) | 19.69 (5.31) | 15.29 (7.27) | 64.74 | 0.01 | <.001 | <.001 |
| Silhouettes | 10.80 (2.29) | 9.90 (2.31) | 7.60 (2.87) | 145.30 | <.001 | .001 | <.001 |
| **Social Cognition** |  |  |  |  |  |  |  |
| Eye Emotional Recognition Task-Mood | 23.63 (4.64) | 22.35 (4.33) | 18.81 (5.31) | 106.65 | <.001 | <.001 | <.001 |
| Eye Emotional Recognition Task-Gender | 30.11 (2.53) | 30.02 (2.34) | 28.69 (4.02) | 19.15 | <.001 | .331 | <.001 |
| **ADNI cohort** |  |  |  |  |  |  |  |
| ADAS-Cog word recall | 7.51 (1.72) | 5.11 (2.48) | 1.40 (11.86) | 178.32 | < .001 | <.001 | <.001 |
| Trail Making Test-B^b^ | 76.19 (32.89) | 116.90 (66.96) | 199.20 (92.54) | 310.08 | < .001 | <.001 | <.001 |

Test raw scores were provided as mean (SD) and analyzed using non-parametric (Kruskal-Wallis) test as distribution of those variables were not normal distribution. *P* values were compared against a Bonferroni-adjusted given the number of tests per hypothesis:α = .05/number of tests (31), 0.002]. a, the subject is instructed to connect numbered circles while alternating colors; b, the subject is instructed to connect the letters and numbers alternatively; c, the effect size of Kruskal-Wallis test. Abbreviations: CN, cognitively normal controls; MCI, mild cognitive impairment; SD, standard deviation; CN-NORM, Chinese Neuropsychological Normative Project; ADNI, Alzheimer’s Disease Neuroimaging Initiative.

Table S4 Comparisons of neuropsychological *z* scores among CN, MCI and Dementia groups in both CN-NORM and ADNI cohorts

| Instruments and domains | CN  Mean (SD) | MCI  Mean (SD) | Dementia  Mean (SD) | *H^c^* | *P* values | *P* values | |
| --- | --- | --- | --- | --- | --- | --- | --- |
|  |  |  |  |  |  | CN vs MCI | CN vs MCI |
| **CN-NORM cohort** |  |  |  |  |  |  |  |
| **Global cognitive function** |  |  |  |  |  |  |  |
| HKBC | 0.00 (3.93) | -8.21 (8.54) | -27.03 (9.83) | 503.19 | <.001 | <.001 | <.001 |
| **Attention** |  |  |  |  |  |  |  |
| Digit Span-Forward | 0.00 (2.71) | -0.48 (2.80) | -2.07 (3.31) | 50.31 | <.001 | .049 | <.001 |
| Digit Span-Forward Length | 0.00 (7.23) | -0.68 (7.72) | -5.21 (9.79) | 52.74 | <.001 | .286 | <.001 |
| Trail Making Test | 0.00 (2.79) | 1.94 (3.10) | 8.54 (9.08) | 238.36 | <.001 | <.001 | <.001 |
| Trail Making Test-A | 0.00 (2.78) | 2.41 (3.48) | 8.40 (7.93) | 256.54 | <.001 | <.001 | <.001 |
| **Memory** |  |  |  |  |  |  |  |
| Hopkins Verbal Learning Test-1 | 0.00 (4.32) | -2.96 (3.75) | -7.20 (3.6) | 283.43 | <.001 | <.001 | <.001 |
| Hopkins Verbal Learning Test-2 | 0.00 (2.45) | -2.07 (2.34) | -5.85 (2.32) | 391.39 | <.001 | <.001 | <.001 |
| Hopkins Verbal Learning Test-3 | 0.00 (2.36) | -2.19 (2.53) | -6.43 (2.7) | 396.99 | <.001 | <.001 | <.001 |
| Hopkins Verbal Learning Test-5 minutes Recall | 0.00 (2.69) | -4.19 (4.04) | -9.76 (2.16) | 468.89 | <.001 | <.001 | <.001 |
| Hopkins Verbal Learning Test-20 minutes Recall | 0.00 (2.20) | -3.16 (3.12) | -7.04 (1.49) | 447.50 | <.001 | <.001 | <.001 |
| Brief Visual Memory Test-1 | 0.00 (3.37) | -2.28 (2.99) | -4.78 (1.38) | 283.70 | <.001 | <.001 | <.001 |
| Brief Visual Memory Test-2 | 0.00 (2.47) | -1.93 (2.49) | -4.65 (1.34) | 339.02 | <.001 | <.001 | <.001 |
| Brief Visual Memory Test-3 | 0.00 (2.46) | -2.62 (3.46) | -6.88 (1.69) | 391.76 | <.001 | <.001 | <.001 |
| Brief Visual Memory Test-30 minutes Recall | 0.00 (2.44) | -2.61 (3.19) | -6.55 (1.34) | 410.58 | <.001 | <.001 | <.001 |
| Logic Memory-1 | 0.00 (1.00) | -0.75 (0.69) | -1.64 (0.52) | 358.11 | <.001 | <.001 | <.001 |
| Logic Memory-2 | 0.00 (6.58) | -5.09 (4.12) | -11.19 (2.72) | 420.79 | <.001 | <.001 | <.001 |
| Logic Memory-30 minutes Recall-1 | 0.00 (1.00) | -0.92 (0.76) | -1.87 (0.26) | 446.80 | <.001 | <.001 | <.001 |
| Logic Memory-30 minutes Recall-2 | 0.00 (6.51) | -6.03 (4.93) | -12.54 (1.84) | 455.63 | <.001 | <.001 | <.001 |
| **Executive Function** |  |  |  |  |  |  |  |
| Trail Making Test-B^a^ | 0.00 (2.80) | 3.25 (2.94) | 6.74 (3.5) | 344.27 | <.001 | <.001 | <.001 |
| Digit Span-Backward | 0.00 (2.95) | -1.10 (2.36) | -3.17 (2.55) | 144.75 | <.001 | <.001 | <.001 |
| Digit Span-Backward Length | 0.00 (4.82) | -1.14 (4.24) | -5.13 (4.58) | 150.64 | <.001 | <.001 | <.001 |
| Digit Symbol Substitute Test | 0.00 (2.89) | -2.32 (2.25) | -5.43 (2.57) | 339.65 | <.001 | <.001 | <.001 |
| Stroop Word Test | 0.00 (2.68) | -1.22 (2.43) | -4.30 (3.21) | 200.71 | <.001 | <.001 | <.001 |
| Stroop Color Test | 0.00 (2.46) | -0.89 (2.08) | -3.82 (2.38) | 224.21 | <.001 | <.001 | <.001 |
| Stroop color-Word Test | 0.00 (2.70) | -1.73 (2.31) | -4.62 (1.81) | 310.18 | <.001 | <.001 | <.001 |
| **Language** |  |  |  |  |  |  |  |
| Animal Naming | 0.00 (4.43) | -2.91 (3.91) | -7.67 (3.93) | 284.75 | <.001 | <.001 | <.001 |
| Boston Naming Test | 0.00 (1.84) | -0.56 (1.85) | -2.62 (2.5) | 142.63 | <.001 | <.001 | <.001 |
| **Visuospatial function** |  |  |  |  |  |  |  |
| Clock Drawing Test | 0.00 (3.30) | -1.00 (4.33) | -7.85 (7.11) | 189.81 | <.001 | 0.010 | <.001 |
| Line Orientation Test | 0.00 (2.57) | -0.75 (2.59) | -2.90 (3.54) | 94.94 | <.001 | <.001 | <.001 |
| Silhouettes | 0.00 (2.82) | -1.11 (2.85) | -3.94 (3.54) | 150.29 | <.001 | <.001 | <.001 |
| **Social Cognition** |  |  |  |  |  |  |  |
| Eye Emotional Recognition Task-Mood | 0.00 (4.41) | -1.21 (4.11) | -4.58 (5.04) | 123.07 | <.001 | <.001 | <.001 |
| Eye Emotional Recognition Task-Gender | 0.00 (6.08) | -0.20 (5.61) | -3.39 (9.65) | 21.00 | <.001 | 0.234 | <.001 |
| **ADNI cohort** |  |  |  |  |  |  |  |
| ADAS-Cog word recall | -1.54 (2.17) | -4.57 (3.12) | -9.24 (2.34) | 178.32 | < .001 | < .001 | < .001 |
| Trail Making Test-B^b^ | -1.51 (1.64) | 0.52 (3.34) | 4.62 (4.61) | 310.08 | < .001 | < .001 | < .001 |

Test *z* scores were provided as mean (SD) and analyzed using non-parametric (Kruskal-Wallis) test as distribution of those variables were not normal distribution. *P* values were compared against a Bonferroni-adjusted given the number of tests per hypothesis: α = .05/number of tests (31), 0.002. a, the subject is instructed to connect numbered circles while alternating colors; b, the subject is instructed to connect the letters and numbers alternatively; c, the effect size of Kruskal-Wallis test. Abbreviations: CN, cognitively normal controls; MCI, mild cognitive impairment; SD, standard deviation; CN-NORM, Chinese Neuropsychological Normative Project; ADNI, Alzheimer’s Disease Neuroimaging Initiative.

Table S5. Cognitive profiles over six cognitive domains by CNCB battery among CN, MCI and dementia group

| Variables | CN  mean (SD) | MCI  mean (SD) | Dementia  mean (SD) | CN vs. MCI | | | MCI vs. Dementia | | |
| --- | --- | --- | --- | --- | --- | --- | --- | --- | --- |
|  |  |  |  | *t* | *P* values | Cohen's *d* | *t* | *P* values | Cohen's *d* |
| Memory | 0.00 (2.15) | -2.83 (2.10) | -6.65 (1.21) | 17.43 | < .001 | 1.33 | -22.71 | < .001 | 2.23 |
| Attention | 0.00 (2.78) | -1.37 (3.16) | -6.06 (5.69) | 4.40 | < .001 | 0.46 | -9.92 | < .001 | 0.17 |
| Executive function | 0.00 ( 2.04) | -1.66 (1.86) | -4.75 (2.11) | 11.12 | < .001 | 0.85 | -17.19 | < .001 | 1.55 |
| Language | 0.00 (2.66) | -1.73 (2.45) | -5.14 (2.64) | 8.84 | < .001 | 0.68 | -14.72 | < .001 | 1.33 |
| Visuospatial function | 0.00 (2.07) | -0.95 (2.23) | -4.89 (3.66) | 5.80 | < .001 | 0.44 | -15.16 | < .001 | 1.30 |
| Social cognition | 0.00 (4.41) | -0.71 (4.13) | -3.99 (6.56) | 2.16 | .031 | 0.17 | -6.95 | < .001 | 0.60 |

Variables were provided as mean (SD). Comparative groups *P* values between two groups were determined by *t* test. Effect size calculated and transformed into Cohen’s d. *P* values were compared against a Bonferroni-adjusted given the number of tests per hypothesis: α = .05/number of tests (6), .008. Abbreviations: CN, cognitively normal controls; MCI, mild cognitive impairment; CNCB, Chinese Neuropsychological Consensus Battery.

Table S6 Feature selection results for discriminating MCI from CN by backward stepwise selection in CN-NORM cohort

| Candidate Variables and order | β | Standardized β | Wald *χ*^2^ | *P* value |
| --- | --- | --- | --- | --- |
| const | -1.31 | -0.10 | 97.97 | < .001 |
| Trail Making Test-B | 0.33 | 0.01 | 60.57 | < .001 |
| Stroop Color test | 0.28 | 0.01 | 19.44 | < .001 |
| Brief Visual Memory Test-30 | -0.18 | 0.00 | 19.44 | < .001 |
| Hopkins Verbal Learning Test | -0.26 | -0.01 | 48.44 | < .001 |
| Animal Naming | -0.07 | 0.00 | 5.91 | .02 |
| Digit Span-Backward Length | 0.06 | 0.00 | 6.06 | .01 |
| Stroop Color-Word Test | -0.14 | 0.00 | 5.98 | .01 |

a, the subject is instructed to connect numbered circles while alternating colors. Abbreviations: CN, cognitively normal controls; MCI, mild cognitive impairment; CN-NORM, Chinese Neuropsychological Normative Project.

Table S7 Feature selection results for discriminating MCI from CN by logistic regression and SelectKBest in CN-NORM cohort

|  | AUC | | | | | Selected K Best | | |
| --- | --- | --- | --- | --- | --- | --- | --- | --- |
| Order | Variable | Fold 0 | Fold 1 | Fold 2 | Mean | Variable | *F* Scores | *P* value |
| 1 | Hopkins Verbal Learning Test-5 minutes Recall | 0.80 | 0.78 | 0.81 | 0.80 | Hopkins Verbal Learning Test-5 minutes Recall | 258.70 | < .001 |
| 2 | Trail Making Test-B | 0.76 | 0.81 | 0.77 | 0.78 | Hopkins Verbal Learning Test-20 minutes Recall | 238.00 | < .001 |
| 3 | Hopkins Verbal Learning Test-20 minutes Recall | 0.77 | 0.77 | 0.80 | 0.78 | Trail Making Test-B | 218.70 | < .001 |
| 4 | Logic Memory-30 minutes Recall-1 | 0.79 | 0.78 | 0.75 | 0.77 | Logic Memory-30 minutes Recall-2 | 184.60 | < .001 |
| 5 | Logic Memory-30 minutes Recall-2 | 0.78 | 0.78 | 0.76 | 0.77 | Logic Memory-30 minutes Recall-1 | 182.80 | < .001 |
| 6 | Logic Memory-2 | 0.76 | 0.76 | 0.72 | 0.75 | Brief Visual Memory Test-30 minutes Recall | 145.80 | < .001 |
| 7 | Digit Symbol Substitute Test | 0.72 | 0.79 | 0.70 | 0.74 | Logic Memory-2 | 144.40 | < .001 |
| 8 | Logic Memory-1 | 0.76 | 0.73 | 0.69 | 0.73 | Hopkins Verbal Learning Test-3 | 137.60 | < .001 |
| 9 | Brief Visual Memory Test-30 minutes Recall | 0.73 | 0.75 | 0.72 | 0.73 | Digit Symbol Substitute Test | 135.80 | < .001 |
| 10 | Trail Making Test-A | 0.75 | 0.77 | 0.67 | 0.73 | Brief Visual Memory Test-3 | 132.50 | < .001 |
| 11 | Hopkins Verbal Learning Test-3 | 0.73 | 0.71 | 0.75 | 0.73 | Logic Memory-1 | 129.50 | < .001 |
| 12 | Brief Visual Memory Test-3 | 0.73 | 0.73 | 0.69 | 0.72 | Hopkins Verbal Learning Test-2 | 127.70 | < .001 |
| 13 | Hopkins Verbal Learning Test-2 | 0.69 | 0.73 | 0.75 | 0.72 | Brief Visual Memory Test-2 | 103.50 | < .001 |
| 14 | Brief Visual Memory Test-2 | 0.72 | 0.73 | 0.68 | 0.71 | Trail Making Test-A | 101.40 | < .001 |
| 15 | Brief Visual Memory Test-1 | 0.70 | 0.67 | 0.73 | 0.70 | Hopkins Verbal Learning Test-1 | 91.10 | < .001 |
| 16 | Trail Making test | 0.68 | 0.73 | 0.69 | 0.70 | Brief Visual Memory Test-1 | 87.20 | < .001 |
| 17 | Animal Naming | 0.70 | 0.72 | 0.65 | 0.69 | Animal Naming | 82.50 | < .001 |
| 18 | Hopkins Verbal Learning Test-1 | 0.68 | 0.67 | 0.72 | 0.69 | Stroop Color-Word Test | 80.80 | < .001 |
| 19 | Stroop Color-Word Test | 0.65 | 0.72 | 0.67 | 0.68 | Trail Making Test | 74.00 | < .001 |
| 20 | Stroop Word Test | 0.63 | 0.67 | 0.59 | 0.63 | Stroop Words Test | 38.40 | < .001 |
| 21 | Stroop color Test | 0.60 | 0.64 | 0.59 | 0.61 | Digit Span-Backward | 28.60 | < .001 |
| 22 | Digit Span-Backward | 0.61 | 0.65 | 0.58 | 0.61 | Silhouettes | 26.10 | < .001 |
| 23 | Silhouettes | 0.60 | 0.65 | 0.58 | 0.61 | Stroop Color Test | 25.90 | < .001 |
| 24 | Eye Emotional Recognition Task-Mood | 0.61 | 0.61 | 0.57 | 0.60 | Boston Naming Test | 15.60 | < .001 |
| 25 | Boston Naming Test | 0.59 | 0.61 | 0.57 | 0.59 | Line Orientation Test | 14.60 | < .001 |
| 26 | Line Orientation Test | 0.61 | 0.56 | 0.58 | 0.58 | Eye Emotional Recognition Task-Mood | 13.80 | < .001 |
| 27 | Digit Span-Backward Length | 0.59 | 0.58 | 0.54 | 0.57 | Clock Drawing Test | 11.60 | .001 |
| 28 | Clock Drawing Test | 0.54 | 0.57 | 0.56 | 0.55 | Digit Span-Backward Length | 10.70 | .001 |
| 29 | Digit Span-Forward | 0.55 | 0.56 | 0.52 | 0.54 | Digit Span-Forward | 5.10 | .025 |
| 30 | Digit Span-Forward Length | 0.54 | 0.51 | 0.52 | 0.52 | Digit Span-Forward Length | 1.40 | .237 |
| 31 | Eye Emotional Recognition Task-Gender | 0.49 | 0.43 | 0.52 | 0.48 | Eye Emotional Recognition Task-Gender | 0.20 | .662 |

a, the subject is instructed to connect numbered circles while alternating colors. *P* values were compared against a Bonferroni-adjusted given the number of tests per hypothesis: one for testing biomarkers against a clinical diagnosis [α = .05/number of tests (31), .002]. Abbreviations: CN, cognitively normal controls; MCI, mild cognitive impairment; AUC, area under the curve; CN-NORM, Chinese Neuropsychological Normative Project.

Table S8 The performance of each model in CN-NORM cohort and ADNI cohort by LR and SVC, respectively

|  | AUC (95%CI) | *P* value  (DeLong test) | Sensitivity (95%CI) | Specificity  (95%CI) | PPV  (95%CI) | NPV  (95%CI) |
| --- | --- | --- | --- | --- | --- | --- |
| **Discrimination by LR (MCI vs CN)** | | | | | | |
| HKBC | 0.80 (0.74-0.86) | NA | 0.72 (0.56-0.88) | 0.81 (0.63-0.99) | 0.76 (0.72-0.80) | 0.74 (0.72-0.76) |
| Model 1 | 0.87 (0.85-0.89) | <0.001 | 0.79 (0.73-0.85) | 0.80 (0.70-0.90) | 0.79 (0.69-0.89) | 0.77 (0.75-0.79) |
| Model 2 | 0.86 (0.84-0.88) | 0.004 | 0.79 (0.69-0.89) | 0.79 (0.63-0.95) | 0.79 (0.69-0.89) | 0.77 (0.73-0.81) |
| Model 3 | 0.83 (0.81-0.85) | 0.134 | 0.70 (0.66-0.74) | 0.83 (0.77-0.89) | 0.75 (0.71-0.79) | 0.75 (0.73-0.77) |
| Model 4 | 0.86 (0.84-0.88) | 0.009 | 0.73 (0.53-0.93) | 0.83 (0.65-1.01) | 0.77 (0.65-0.89) | 0.75 (0.71-0.79) |
| Model 5 | 0.85 (0.83-0.87) | 0.011 | 0.82 (0.72-0.92) | 0.72 (0.52-0.92) | 0.78 (0.68-0.88) | 0.73 (0.71-0.75) |
| Model 5 (ADNI) | 0.81 (0.81-0.81) | NA | 0.76 (0.76-0.76) | 0.76 (0.74-0.78) | 0.73 (0.71-0.75) | 0.78 (0.78-0.78) |
| **Discrimination by SVC (MCI vs CN)** | | | | | | |
| HKBC | 0.80 (0.74-0.86) | NA | 0.72 (0.56-0.88) | 0.81 (0.63-0.99) | 0.82 (0.74-0.90) | 0.73 (0.71-0.75) |
| Model 1 | 0.86 (0.84-0.88) | 0.001 | 0.72 (0.54-0.90) | 0.86 (0.70-1.02 | 0.80 (0.70-0.90) | 0.76 (0.72-0.80) |
| Model 2 | 0.86 (0.84-0.88) | 0.004 | 0.74 (0.58-0.90) | 0.84 (0.72-0.96) | 0.79 (0.71-0.87) | 0.76 (0.72-0.80) |
| Model 3 | 0.83 (0.81-0.85) | 0.125 | 0.71 (0.65-0.77) | 0.82 (0.74-0.90) | 0.78 (0.70-0.86) | 0.74 (0.72-0.76) |
| Model 4 | 0.85 (0.83-0.87) | 0.010 | 0.79 (0.73-0.85) | 0.76 (0.66-0.86) | 0.78 (0.66-0.90) | 0.74 (0.72-0.76) |
| Model 5 | 0.85 (0.83-0.87) | 0.014 | 0.83 (0.75-0.91) | 0.72 (0.62-0.82) | 0.77 (0.67-0.87) | 0.74 (0.72-0.76) |
| Model 5 (ADNI) | 0.81 (0.81-0.81) | NA | 0.75 (0.73-0.77) | 0.76 (0.76-0.76) | 0.73 (0.71-0.75) | 0.78 (0.78-0.78) |
| **Discrimination by LR (Dementia vs MCI)** | | | | | | |
| HKBC | 0.92 (0.90-0.94) | NA | 0.82 (0.74-0.90) | 0.88 (0.82-0.94) | 0.82 (0.72-0.92) | 0.88 (0.86-0.90) |
| Model 1 | 0.94 (0.92-0.96) | 0.052 | 0.89 (0.79-0.99) | 0.88 (0.80-0.96) | 0.82 (0.74-0.90) | 0.91 (0.87-0.95) |
| Model 2 | 0.93 (0.91-0.95) | 0.301 | 0.91 (0.81-1.01) | 0.83 (0.71-0.95) | 0.80 (0.74-0.86) | 0.91 (0.87-0.95) |
| Model 3 | 0.92 (0.90-0.94) | 0.572 | 0.89 (0.79-0.99) | 0.83 (0.79-0.87) | 0.76 (0.76-0.76) | 0.90 (0.86-0.94) |
| Model 4 | 0.91 (0.89-0.93) | 0.446 | 0.91 (0.87-0.95) | 0.78 (0.70-0.86) | 0.74 (0.72-0.76) | 0.87 (0.79-0.95) |
| Model 5 | 0.90 (0.88-0.92) | 0.338 | 0.87 (0.83-0.91) | 0.81 (0.75-0.87) | 0.74 (0.70-0.78) | 0.86 (0.78-0.94) |
| Model 5 (ADNI) | 0.89 (0.89-0.89) | NA | 0.85 (0.83-0.87) | 0.84 (0.82-0.86) | 0.73 (0.71-0.75) | 0.84 (0.82-0.86) |
| **Discrimination by SVC (Dementia vs MCI)** | | | | | | |
| HKBC | 0.92 (0.90-0.94) | NA | 0.82 (0.74-0.90) | 0.88 (0.82-0.94) | 0.82 (0.72-0.92) | 0.88 (0.86-0.90) |
| Model 1 | 0.94 (0.92-0.96) | 0.089 | 0.89 (0.79-0.99) | 0.87 (0.81-0.93) | 0.83 (0.77-0.89) | 0.91 (0.89-0.93) |
| Model 2 | 0.93 (0.91-0.95) | 0.372 | 0.88 (0.82-0.94) | 0.88 (0.86-0.90) | 0.79 (0.77-0.81) | 0.93 (0.89-0.97) |
| Model 3 | 0.91 (0.87-0.95) | 0.556 | 0.90 (0.84-0.96) | 0.83 (0.81-0.85) | 0.76 (0.76-0.76) | 0.93 (0.91-0.95) |
| Model 4 | 0.90 (0.88-0.92) | 0.358 | 0.90 (0.88-0.92) | 0.79 (0.71-0.87) | 0.73 (0.69-0.77) | 0.88 (0.82-0.94) |
| Model 5 | 0.89 (0.87-0.91) | 0.236 | 0.87 (0.83-0.91) | 0.82 (0.76-0.88) | 0.73 (0.65-0.81) | 0.87 (0.79-0.95) |
| Model 5 (ADNI) | 0.89 (0.89-0.89) | NA | 0.86 (0.82-0.90) | 0.84 (0.80-0.88) | 0.73 (0.69-0.77) | 0.85 (0.83-0.87) |

Model 5 (ADNI), The performance of Model 5 in ADNI cohort. *P* < .05 indicated the statistical significance of the difference. Abbreviations: AUC, area under the curve. CI, confidence interval; LR, logistic regression; SVC, Support Vector Classification; PPV, Positive Predictive Value; NPV, Negative predictive value; CN, cognitively normal controls; MCI, mild cognitive impairment; CN-NORM, Chinese Neuropsychological Normative Project; ADNI, Alzheimer’s Disease Neuroimaging Initiative; NA, not applicable.


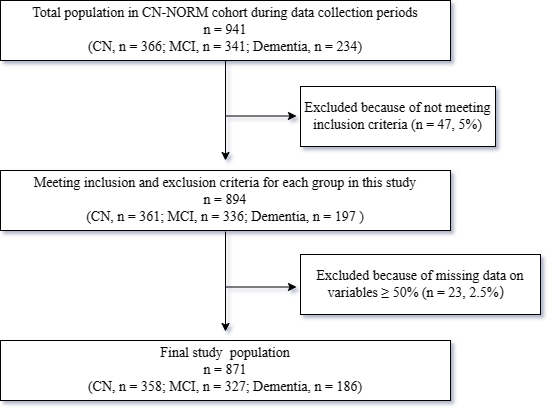


**eFigure 1. Flow-chart of study population in CN-NORM cohort**

Abbreviations: CN, cognitively normal controls; MCI, mild cognitive impairment; CN-NORM, Chinese Neuropsychological Normative Project.

**eReferences**

1. Yaoxian G. Manual for the Wechsler Adult Intelligence Scale: Revised for China (WAIS-RC). Changsha, Hunan, China: Hunan Medical College; 1982.

2. Reitan RM. The relation of the trail making test to organic brain damage. Journal of consulting psychology 1955; 19(5): 393-4.

3. Yaoxian G. Manual for the Wechsler Memory Scale: Revised for China (WMS-RC). Hunan, Changsha, China: Hunan Medical College; 1989.

4. Ralph H. B. Benedict DS, Lowell Groninger, and Jason Brandt. Hopkins Verbal Learning Test – Revised: Normative Data and Analysis of Inter-Form and Test-Retest Reliability. Clinical Neuropsychologist 1998; 12(1): pp43-55.

5. Ralph H.B. Benedict LG, David Schretlen, Melissa Dobraski, Barnett Shpritz. Revision of the brief visuospatial memory test: Studies of normal performance, reliability, and validity. Psychological Assessment 1996; 8(2): 145-53.

6. Stroop JR. Studies of interference in serial verbal reactions. Journal of Experimental Psychology 1935; 18: 643-62.

7. Morris JC, Heyman A, Mohs RC, et al. The Consortium to Establish a Registry for Alzheimer's Disease (CERAD). Part I. Clinical and neuropsychological assessment of Alzheimer's disease. Neurology 1989; 39(9): 1159-65.

8. Guo Qihao HZ, Shi Weixiong, Sun Yinmin, Lv Chuanzhen. Boston Naming Test in Chinese Elderly, Patient with Mild Cognitive Impairment and Alzheimer's Dementia. CHINESE MENTAL HEALTH JOURNAL 2006; 20(2): 81-4.

9. Benton AL. Contributions to Neuropsychological Assessment: A Clinical Manual. New York: Oxford University Press; 1983.

10. Huang L, Chen KL, Lin BY, et al. An abbreviated version of Silhouettes test: a brief validated mild cognitive impairment screening tool. International psychogeriatrics 2019; 31(6): 849-56.

11. Yesavage JA, Brink TL, Rose TL, et al. Development and validation of a geriatric depression screening scale: a preliminary report. J Psychiatr Res 1982; 17(1): 37-49.

12. Chan AC. Clinical validation of the Geriatric Depression Scale (GDS): Chinese version. J Aging Health 1996; 8(2): 238-53.
